# Non-hematopoietic erythropoietin splice variant is produced in the diseased human brain and confers neuroprotection

**DOI:** 10.3389/fncel.2025.1677505

**Published:** 2026-01-12

**Authors:** Theresa Hartung, Dorette Freyer, Anne Zemella, Helena Radbruch, January Weiner, Jasmin Jamal El-Din, Andreas Meisel, Josef Priller

**Affiliations:** 1 Department of Neurology with Experimental Neurology, Charité - Universitätsmedizin Berlin, Berlin, Germany; 2 Berlin Institute of Health at Charité University Medicine Berlin Anna-Louisa-Karsch Straße, Berlin, Germany; 3 Department of Neuropsychiatry and Laboratory of Molecular Psychiatry, Charité - Universitätsmedizin Berlin and DZNE, Berlin, Germany; 4 Fraunhofer Institute for Cell Therapy and Immunology, Branch Bioanalytics and Bioprocesses (IZI-BB), Potsdam, Germany; 5 Department of Neuropathology, Charité - Universitätsmedizin Berlin, Freie Universität Berlin, Humboldt-Universität zu Berlin, Berlin, Germany; 6 Core Unit Bioinformatics, Berlin Institute of Health at Charité - Universitätsmedizin Berlin, Berlin, Germany; 7 Zentrum für Schlaganfallforschung Berlin, Charité - Universitätsmedizin Berlin, Berlin, Germany; 8 Neurowissenschaftliches Forschungszentrum Berlin, Charité - Universitätsmedizin Berlin, Berlin, Germany; 9 Department of Psychiatry and Psychotherapy, and DZPG, Technische Universität München, Munich, Germany; 10 The University of Edinburgh Centre of Clinical Brain Science, Edinburgh, United Kingdom

**Keywords:** alternative splicing, cell-free glycoprotein synthesis, erythropoietin, *in situ* hybridization, neuroprotection, oxygen glucose deprivation, pluripotent stem cells

## Abstract

Erythropoietin (EPO) is a pleiotropic cytokine with important functions in neuronal development and neuroprotection, but hematopoietic effects limit the therapeutic application of EPO in neurological diseases. We discovered human endogenous EPO splice variants that are non-hematopoietic but cytoprotective. Here, we demonstrate at the single-cell level that an alternative splice variant lacking exon 3 (hS3) is expressed in the human brain and is upregulated above EPO mRNA levels in ischemic and inflammatory neurological diseases. Conversely, hS3 mRNA expression is reduced below EPO levels in neurodegenerative disease. In an oxygen–glucose deprivation (OGD) model of ischemia, a single dose of cell-free synthesized constant glycosylated active hS3 protects neuronal cultures derived from human induced pluripotent stem cells (hiPSC) and human embryonic stem cells (hESC) more effectively than EPO. We identify the D-helix as a key functional domain of hS3 and demonstrate that the neuroprotective effect is enhanced by PD29, a novel small peptide derived from the D-helix of hS3. Long-term hS3 administration increases the neuroprotective effects in the OGD model by dose-dependent differential expression of apoptosis-related protein-coding genes and long non-coding RNAs (lncRNAs). In addition, our results suggest that hS3 induces early cell cycle inhibition without impairing differentiation of hiPSC and hESC into neuronal subtypes. In conclusion, EPO splice variant hS3 is part of the endogenous neuroprotective system in the human brain with significant therapeutic potential.

## 1 Introduction

Erythropoietin (EPO) is a pleiotropic cytokine involved in cell differentiation, proliferation and survival (Metcalf, 2008). Anti-apoptotic effects of EPO have also been observed in response to hypoxia within the central nervous system (CNS) (Noguchi et al., 2007). EPO expression can be independently induced by hypoxic or inflammatory stimuli through activation of the hypoxia-inducible factor 1 (HIF-1) pathway (Semenza, 2001; Mi et al., 2008; Oh et al., 2008), contributing to self-repair mechanisms. Endogenous EPO has been shown to inhibit neuronal apoptosis and promote neurogenesis, acting as a paracrine and autocrine mediator within the CNS (Shingo et al., 2001; Ruscher et al., 2002; Wakhloo et al., 2020). In addition, endogenous EPO exhibits immunomodulatory effects by reducing the proliferation of conventional T cells without inducing apoptosis, while simultaneously promoting proliferation of regulatory T cells (Treg) and decreasing B cell numbers (Cravedi et al., 2014; Purroy et al., 2017; Peng et al., 2020), which may be beneficial in chronic inflammatory diseases of the CNS, e.g., multiple sclerosis (MS) (Baecher-Allan et al., 2018). However, the anti-apoptotic and proliferative effects of EPO are not cell type-specific, and evidence indicates that EPO can directly enhance the proliferation of cancer cells. EPO has been identified as a negative prognostic marker in human liver and renal cancers, where its expression is induced by hypoxic conditions associated with tumor growth and severe tumor-induced anemia. Additionally, EPO can indirectly promote cancer progression by inhibiting chemotherapy-induced cell death, thereby contributing to treatment resistance (Michael et al., 2007; Ribatti et al., 2007; Debeljak et al., 2014).

While EPO expression is restricted to a limited number of cell types, mainly astrocytes and neurons in the CNS and macrophages and plasma cells in the immune system, EPO receptors (EPOR) are expressed ubiquitously, facilitating EPOs pleiotropic effects (Hodge et al., 2019; Sjostedt et al., 2020). To date, four distinct receptor complexes have been identified, each contributing to different biological functions (Ma et al., 2022; Knorr et al., 2023). First, the homodimeric EPOR, composed of two EPOR units ((EPOR)_2_), is primarily responsible for transmitting erythropoietic signals but has also been implicated in cytoprotection in neuronal models (Um et al., 2007) Second, the heterodimeric receptor complex, consisting of one EPOR unit and one β-common receptor subunit (EPOR/ßcR), primarily mediates erythropoiesis-independent, tissue-protective effects. Third, the ephrin receptor B4 (EphB4) has been implicated in promoting tumor growth and regulating adult neurogenesis and gliogenesis. However, since the recent identification of cytokine receptor-like factor 3 (CRLF3) as a specified neuroprotective EPO receptor (Knorr et al., 2023), both EPOR₂- and EPOR/βcR-dependent neuroprotective effects may need to be re-evaluated, as CRLF3 could mediate part of this signaling. So far, receptor-specific knockout combination studies that could clearly distinguish between EPOR₂-, EPOR/βcR-, and CRLF3-dependent cytoprotection are lacking, leaving receptor utilization context-dependent and not yet fully resolved.

In neurological diseases, EPO is induced under hypoxic conditions, e.g., ischemic stroke, and inflammatory conditions, e.g., experimental autoimmune encephalomyelitis (EAE) (Mengozzi et al., 2008). In contrast, EPO levels decrease with aging and are further reduced in neurodegenerative diseases, including amyotrophic lateral sclerosis (ALS) (Brettschneider et al., 2007; Li et al., 2016; Torpel et al., 2019). Since EPO can cross the blood–brain barrier (BBB) (Brines et al., 2000), peripheral administration of EPO has been investigated as a potential neuroprotective treatment in neurological disease models for stroke (Zhang et al., 2006), multiple sclerosis (MS) (Cervellini et al., 2013), and ALS (Grunfeld et al., 2007). However, clinical trials to date have yielded inconsistent and controversial outcomes (Ehrenreich et al., 2007; Ehrenreich et al., 2009; Asadi et al., 2013; Tsai et al., 2015; Schreiber et al., 2017). The clinical application of peripherally administered EPO is significantly limited by its hematopoietic side effects, including an increased risk of thrombosis. To overcome these limitations, research has focused on developing non-hematopoietic full-length EPO derivatives, including asialoerythropoietin (Erbayraktar et al., 2003), carbamylated EPO (CEPO) (Leist et al., 2004), and nasal NeuroEPO (Santos-Morales et al., 2017). Despite their promising neuroprotective potential, these derivatives face substantial challenges, including structural instability, lack of essential post-transcriptional modifications, and immunogenicity, thereby restricting their clinical applicability (Brines, 2014).

Complex post-translational modification, including glycosylation, are essential for protein bioactivity by affecting plasma clearance and immunogenicity. Consequently, glycoengineering has gained increasing relevance in the optimization of therapeutic proteins (Chen et al., 2022). A highly efficient alternative to time-consuming cell-based production is cell-free glycoprotein synthesis, which has recently been established for heavily glycosylated EPO (Zemella et al., 2018). This rapid, high-yield method holds significant promise for future drug development, including antibody production and vaccine development, with clinical trials already underway (Dondapati et al., 2020). Cell-free glycoprotein synthesis has the potential to improve the production of non-hematopoietic EPO derivatives.

Another strategy leveraging the exclusively neuroprotective function of EPO is the development of short synthetic peptides derived from the neuroprotective domain of EPO. These small peptides have significant potential for clinical translation due to their ability to more easily cross the BBB, exhibit high receptor affinity, possess low immunogenicity, and offer cost-effective production (Peng et al., 2020). Like other class I cytokines, EPO adopts a four-helix bundle structure consisting of four *α*-helices (A, B, C, and D) (Boulay et al., 2003). Critical sites for bioactivity and receptor binding are located within these helices and the AB-loop (Elliott et al., 1997; Brines et al., 2004), which have served as templates for the development of short EPO-derived peptide candidates. Among the peptides investigated to date are Epopeptide AB (Nagao et al., 2005), JM-4 (Yuan et al., 2015), Helix B surface peptide (HBSP/ARA290) (Brines et al., 2008), Cyclic Helix B peptide (CHBP) (Yang et al., 2014), Epotris (Pankratova et al., 2010), ML1-h3 (Cho et al., 2022), and NL100 (Dmytriyeva et al., 2019). These peptides are primarily derived from the AB-loop, B-helix, or C-helix and have demonstrated neuroprotective effects *in vitro* and *in vivo*, mediated through interactions with either the homodimeric EPOR/EPOR or heterodimeric EPOR/βCR receptor complexes. Although HBSP has shown clinical efficacy in improving neuropathy associated with sarcoidosis, clinical trials for other EPO-derived peptides remain scarce, possibly due to concerns that activation of the EPOR pathway may also contribute to cancer progression (Peng et al., 2020). Notably, peptides derived from the D-helix have yet to be investigated.

The human endogenous non-hematopoietic EPO alternative splice variant hS3 (also known as hEPOΔ3 or EV-3), initially identified in the human kidney and fetal brain (Meisel et al., 2006; Bonnas, 2009), is characterized by deletion of exon 3. Alike EPO, hS3 has cytoprotective effects in murine in vitro and in vivo models, in human iPSC-derived neurons and is upregulated in human liver cirrhosis and renal cell carcinoma (Bonnas, 2009; Bonnas et al., 2017; Knorr et al., 2023). In contrast to EPO, hS3 is believed to bind exclusively to the neuroprotective receptor, CRLF3, with no evidence of interaction with classical hematopoietic EPO receptors (Bonnas et al., 2017). Regarding its immunomodulatory properties, hS3 more strongly induces anti-inflammatory cytokines and suppresses proinflammatory cytokines than EPO, underscoring its therapeutic potential in inflammatory diseases of the CNS (Meisel et al., 2006). However, to date, no studies have investigated hS3 expression in the adult human brain or in neurological diseases.

Here, we aim to characterize hS3 mRNA expression in the human brain in various diseases of the central nervous system. In addition, we want to investigate the functions of hS3, in human neuronal development and in particular its potential neuroprotective and neuroregenerative properties in stroke models.

## 2 Materials and methods

### 2.1 Human tissue and human cell lines

Human 5 μm formalin-fixed paraffin-embedded (FFPE) brain sections were obtained from the BrainBank of the Department of Neuropathology, Charité, with full pre-mortem consent from donors and ethical approval for both the BrainBank collection (EA1/144/13) and the specific utilization of samples in this study (EA1/205/21) granted by the Charité Ethics Committee. hiPSC-derived neurons were differentiated from the established and fully characterized human reference cell line BIHi005-A. This cell line was obtained from the Berlin Institute of Health, Stem Cell Core Facility, which holds donor consent and full ethical approval. The BIHi005-A cell line is publicly registered in the Human Pluripotent Stem Cell Registry.^1^ hESC-derived neurons were differentiated from the established and fully characterized human reference cell line H1 (WA01). The H1 cell line was obtained from the Max Delbrück Center for Molecular Medicine and is registered in the WiCell catalog.^2^ Usage approval for this cell line was granted by the Robert Koch Institute (AZ: 3.04.02/0159). Ethical approval for the use of both cell lines in this study particularly was granted by the Charité Ethics Committee (EA2/278/23).

### 2.2 BaseScope mRNA detection

EPO mRNA consists of five exons, while the human splice variant hS3 is characterized by the deletion of exon 3. To detect EPO and hS3 mRNA in tissue samples, customized BaseScope single oligonucleotide 1ZZ probes were designed by Advanced Cell Diagnostics. The EPO probe targets a 38-base sequence spanning the exon 2 to exon 3 junction (hEPO: NPR-0026806, BA-Hs-EPO-E2E3, positions 646–683, sequence: GAGGCCAAGGAGGCCGAGAATATCACG ACGGGCTGTGC), while the hS3 probe targets a 36-base sequence spanning the exon 2 to exon 4 junction (hS3: NPR-0026807, BA-Hs-EPO-E2E4, positions 647–682, sequence: AGGCCAAGGAGGCCG AGAATATCACGGTCGGGCAGC). Specificity of both probes was validated by aligning the sequences to the human genome using the Basic Local Alignment Search Tool (BLAST), confirming that no off-target sequences were detected. The BaseScope probes are designed as “Z” probe pairs. The lower region of the probe binds the target mRNA, while the upper region contains a preamplifier binding site to facilitate the attachment of multiple amplifiers, thereby enhancing the chromogenic signal. The EPO probe was assigned to channel 2 and was detected using an alkaline phosphatase Fast Red-based reaction, resulting in a red signal. The hS3 probe was assigned to channel 1 and was detected using a horseradish peroxidase-based reaction, producing a green signal. Positive control experiments were initially conducted using previously established CHO cell lines expressing either EPO or hS3 (Bonnas, 2009). The RNAscope Assay for Adherent Cells and the Sample Preparation Technical Note for Cultured Adherent Cells Using the RNAscope 2.5 Chromogenic Assay were followed according to the manufacturer’s protocols, with no adjustments. For human brain sections the BaseScope Duplex Detection Assay for FFPE tissue was performed according to the manual with adjustments, in the following highlighted in bold letters. RNA integrity and hybridization efficiency were verified by running positive and negative control probes in parallel with experimental samples. Positive control probes target the housekeeping genes, peptidylprolyl isomerase B (PPIB, Channel 1, green) and RNA polymerase II subunit A (POLR2A, Channel 2, red). The universal negative control probe targets dihydrodipicolinate reductase (DapB) from *Bacillus subtilis* (accession #EF191515). All incubation steps at 40 °C or 60 °C were conducted using the HybEZ II Oven. FFPE brain sections (5 μm thick, 3 mm in diameter) were baked at 60 °C for 1 h, followed by deparaffinization in fresh xylene for two 5-min intervals and 100% ethanol for two 2-min intervals. After drying at 60 °C for at least 5 min, sections were treated with hydrogen peroxide for 10 min at room temperature and then washed twice in deionized water. Target retrieval was performed by placing the slides in a steamer for 10 s in deionized water, followed by 45 min in 200 mL target retrieval buffer. After washing in deionized water for 15 s and 100% ethanol for 3 min, the sections were dried at 60 °C for at least 5 min. A hydrophobic barrier was applied around the tissue using the ImmEdge barrier pen and allowed to dry completely. Protease treatment was performed using Protease IV for 60 min at 40 °C, after which the sections were washed twice in deionized water. Control and target probes were prewarmed to 40 °C in a water bath before being hybridized to the tissue sections for 2.5 h at 40 °C. Following hybridization, sections were washed twice in wash buffer for 2 min and stored in 5X SSC buffer at room temperature overnight, according to the optional stopping point 3 described in the manual. Amplification and chromogenic detection were performed through a series of amplification steps, with each step followed by two washes in wash buffer for 2 min and removal of excess fluid by flicking the slides. The amplification steps included Amp1 for 30 min at 40 °C, Amp2 for 30 min at 40 °C, Amp3 for 15 min at 40 °C, Amp4 for 30 min at 40 °C, Amp5 for 30 min at 40 °C, Amp6 for 15 min at 40 °C, Amp7 for 60 min at room temperature, and Amp8 for 15 min at room temperature. The Fast Red chromogenic reaction was applied for 10 min at room temperature, protected from light. After additional amplification steps (Amp9 for 15 min at 40 °C, Amp10 for 15 min at 40 °C, Amp11 for 60 min at room temperature, and Amp12 for 15 min at room temperature), the green chromogenic reaction was applied for 10 min at room temperature, protected from light. Slides were briefly immersed in deionized water to stop the reaction. Sections were counterstained for 35 s with Hematoxylin (Gill’s Hematoxylin I, American Master Tech Scientific), briefly rinsed in tap water three times, and dried at 60 °C for at least 60 min before being mounted with VectaMount and air dried overnight. BaseScope signals were visualized using a standard bright-field microscope (Olympus IX83) at 40 × magnification, following the manufacturer’s recommendations (Advanced Cell Diagnostics). The BaseScope assay is validated for single-plane imaging and enables visualization of individual mRNA molecules as discrete puncta without the need for z-stack acquisition.

### 2.3 Cell-free protein synthesis of glycosylated hS3 and EPO with subsequent characterization

Gene sequences coding for hS3 and EPO were modified by introducing an internal ribosomal entry site upstream of the gene. Regulatory elements were introduced in 5’and 3′ sequences. Furthermore, endogenous signal peptide was exchanged by a melittin signal sequence. The sequences were manufactured by *de novo* synthesis (Biocat GmbH, Germany) and ligated into the pUC57-1.8 k vector backbone. Cell-free protein synthesis was conducted using translationally active lysates obtained from cultured *Spodoptera frugiperda 21* (Sf21) cells. Lysate preparation was carried out as previously described (Stech et al., 2012; Thoring et al., 2019). A DNA template coding for hS3 or EPO (60 ng/μL) was added into a reaction mixture consisting of 20% (v/v) lysate, 100 μM amino acids, salts, energy components, and PolyG (10 μM, Biomers, Germany). A detailed protocol for the reaction can be found in Brodel et al. (2013). To assess protein yield through scintillation counting and protein integrity via SDS-PAGE followed by autoradiography, radioactively labeled 14C-leucine (f.c. 30 μM, Perkin Elmer, Germany) was added to the reaction. The 14C-leucine is statistically incorporated into the newly synthesized protein. The reactions were incubated for 3 h at 27 °C, 500 rpm. Quantitative analysis of protein yield of hS3 and EPO was determined by hot trichloroacetic acid (TCA, Carl Roth GmbH, Germany) precipitation followed by liquid scintillation counting (Ramm et al., 2020). Qualitative analysis of protein integrity and glycosylations of hS3 and EPO was performed by autoradiography. Therefore, samples were precipitated in cold acetone, dried and solved in LDS sample buffer (NuPAGE LDS sample buffer, Thermo Fisher Scientific). Afterwards, samples were loaded onto precast SDS-PAGE gels (NuPAGE, 10% Bis–Tris, Thermo Fisher Scientific) and separated for 35 min at 180 V. After staining the gels with simply blue safe stain (Thermo Fisher Scientific), gels were washed again and dried for 60 min at 70 °C (Unigeldryer 3545D, Germany) onto a Whatman paper. Gels were stored at a phosphor screen for 3 days and the protein bands were visualized by phosphor imaging (Amersham Typhoon RGB, GE Healthcare). Deglycosylation assay of N-glycosylated hS3 and EPO by using PNGase F was performed according to the manufacturer’s protocol (NEB, USA). HS3 and EPO were translocated into the lumen of the microsomes. Therefore, the proteins had to be released out of the microsomes for further characterization. Translation mixture was centrifuged at 16,000 xg for 10 min at 4 °C and the pellet was resuspended in PBS containing the detergent n-Dodecyl-*β*-Maltoside (0.2% DDM, Sigma-Aldrich, St. Louis MO, USA) and agitated for 45 min at 1000 rpm. Afterwards, the sample was centrifuged again and the supernatant was collected for cell culture assays. Cell culture assay was performed as previously described (Pouyan et al., 2023). In short, TF-1 cells (Leibnitz-Institut DSMZ, Germany, DSMZ-No. ACC-334) were cultivated in 85% RPMI-1640 (PAN Biotech), 13% FCS (Biochrom), 1% sodium pyruvate (Biowest), 1% penicillin–streptomycin (PAN Biotech) and 5 ng/mL GM-CSF (PeproTech, Germany) at 37 °C and 5% CO2 in a CO2 incubator (Binder, Germany). For the proliferation assay 2.0 × 10^5^ cells were transferred into fresh medium without the growth hormone GM-CSF. HS3, EPO, positive, and negative controls were added at final concentration of 10 and 30 ng/mL. Cell growth was monitored over 4 days by trypan blue staining in a Luna counting chamber (Logos biosystems).

### 2.4 Neural differentiation of healthy hiPSC- and hESC-derived neuronal cultures in miniaturized small-scale systems

Neural progenitor cells (NPCs) were derived from the BIHi005-A hiPSC line and the H1 hESC line as previously described (Chambers et al., 2009; Shi et al., 2012). For primary expansion, 6-well plates were coated with Geltrex (Thermo Fisher, A1413302). Geltrex was initially diluted 1:10 in ice-cold KnockOut DMEM/F-12 (Thermo Fisher, 12,660,012) to create a stock solution, which was then further diluted 1:11 in ice-cold KnockOut DMEM/F-12. Plates were coated with 1 mL per well and incubated at room temperature for 1 h. NPCs were thawed and washed in KnockOut DMEM/F-12 supplemented with 10 μM ROCK inhibitor Y-27632 (STEMCELL Technologies, 72,304). Cells were then resuspended in Neural Expansion Medium (NEM), consisting of a 50:50 mixture of Advanced DMEM/F-12 (Thermo Fisher, 12,634–010) and Neurobasal Medium (Thermo Fisher, 21,103–049), with 1:50 Neural Induction Supplement (Thermo Fisher, A1647701) and 1:100 Penicillin–Streptomycin (Thermo Fisher, 15,140–122), containing 10 μM ROCK inhibitor. Cells were seeded at a density of 5 × 10^5^ cells per well in 6-well plates. Cell counting was performed using a Neubauer counting chamber, counting 4 × 16 squares. Medium was changed daily without ROCK inhibitor until the NPCs reached confluence. NPCs were expanded and passaged twice before initiating differentiation. For differentiation, 7.5 × 10^5^ NPCs were seeded into Geltrex-coated wells of a 6-well plate. The differentiation medium consisted of a 50:50 mixture of Neurobasal Medium and DMEM/F-12 with Glutamax supplement (Thermo Fisher, 31,331–028), with 1:200 N2 Supplement, 1:100 B27 Supplement, 1% Penicillin–Streptomycin, 250 μM dibutyryl cyclic AMP (db-cAMP), 150 μM ascorbic acid, 20 ng/mL brain-derived neurotrophic factor (BDNF), 20 ng/mL glial cell line-derived neurotrophic factor (GDNF), and 2 mM Glutamax. Medium was changed every 2 days. On day 15, cells were replated onto Geltrex-coated 6 well plates at same density. On day 25, cells were replated on 8-well chamber slides (Nunc Lab-Tek II Chamber Slide System, ThermoScientific, 177402PK). The slides were first coated with poly-DL-ornithine hydrobromide (POR) (Merck, P8638-25 mg) diluted in 0.15 mM borate buffer to a final concentration of 0.05 μg/μl, followed by a second coating with laminin (Merck, L2020) diluted in H₂O to a final concentration of 2 μg/cm^2^. Cells were seeded at a density of 0.5 × 10^5^ cells per well. All OGD and immunofluorescence experiments were performed using neuronal cultures grown on 8-well chamber slides. Cultures were maintained in an incubator under a controlled atmosphere at 37 °C, 100% humidity, and 5% CO₂ until the day of the experiment.

### 2.5 OGD in miniaturized small-scale systems as an *in-vitro* stroke model and neuroprotection assay

To assess neuroprotective effects under OGD conditions, hiPSC- and hESC-derived neuronal cultures were subjected to either 48-h pretreatment or continuous treatment with hS3. For 48-h pretreatment, differentiation medium was supplemented with 10 ng/mL, 20 ng/mL, or 30 ng/mL hS3, with the final medium change occurring on differentiation day 27, 48 h prior to OGD. For continuous treatment, the differentiation medium was supplemented with either 10 ng/mL or 20 ng/mL hS3 at each medium change throughout the differentiation period. On differentiation day 29, conditioned medium from each treatment group was collected, and cells were washed with Balanced Salt Solution (BSS) buffer without glucose before being transferred to a hypoxic workstation (Invivo2 400, Ruskinn Technology) set to a predefined atmosphere of 0.3% O₂, 5% CO₂, 0% H₂ (non-reductive atmosphere), 37 °C, and 100% humidity. Inside the hypoxic workstation, BSS buffer was replaced with 24-h preconditioned, deoxygenated, glucose-free BSS buffer, and OGD was performed for 8 h. For the first 4 h, the lid of the chamber slides was removed to allow direct exposure to the hypoxic environment. Control cultures were washed and incubated with BSS buffer containing glucose and maintained under normoxic conditions at 37 °C with 5% CO₂. Immediately after OGD, the treated and control cultures were returned to a 2/3 fresh differentiation medium and 1/3 conditioned medium mixture. After 24 h of reoxygenation cell damage was assessed using a kinetic lactate dehydrogenase (LDH) Assay, based on an established protocol (Freyer and Harms, 2017) with adaptions due to cell type and small-scales applied. In brief, the LDH assay measures cell damage by detecting the release of LDH, an enzyme that catalyzes the reversible conversion of pyruvate and NADH (reduced nicotinamide adenine dinucleotide) to lactate and NAD^+^ (oxidized nicotinamide adenine dinucleotide). The assay relies on the characteristic absorption of NADH at 340 nm, with a decrease in optical density at this wavelength corresponding to the consumption of NADH during the reaction. The concentration of LDH in the culture supernatant was determined using a standard enzyme solution with known LDH activity as a reference. To control for potential confounding factors, such as variations in cell density or metabolic activity between individual wells, normalization of LDH activity was performed by measuring total LDH activity following complete cell lysis using 0.5% Triton X-100 (referred to as “full kill”). The percentage of LDH activity in the supernatant relative to the maximum releasable LDH activity (full kill) provides a proportional measure of cell damage within the individual cell culture well. In contrast to the original protocol, kinetic measurements were performed using 25 μL of culture supernatants, monitored over 20 cycles with 30-s intervals. Maximum releasable LDH activity was determined for each well following incubation with 0.5% Triton X-100 for at least 60 min. All measurements were conducted in duplicates.

### 2.6 Immunofluorescence

For immunofluorescence staining, cells cultured on 8-well chamber slides were fixed with 4% formaldehyde for 20 min at room temperature, washed three times with phosphate-buffered saline, and blocked for 60 min in blocking buffer containing 10% goat serum, 1% bovine serum albumin (BSA), and 0.3% Triton X-100 to reduce nonspecific binding. The following primary antibodies were applied and incubated overnight at 4 °C: mouse monoclonal anti-TuJ1 (1:250, BioLegend, MMS-435P), rabbit monoclonal anti-Ki-67 (1:250, Abcam, ab16667), rabbit monoclonal anti-GFAP (1:250, Abcam, ab68428), mouse monoclonal anti-Olig2 (1:250, Merck, MABN50), mouse monoclonal anti-*α*-S100 beta subunit (1:100, Merck, S2532), and rabbit monoclonal anti-IBA1 (1:200, Abcam, ab178846). After additional washing steps, secondary antibodies (Alexa Fluor Donkey anti rabbit 568, 1:500, A10042, Donkey anti mouse 488, 1:1000, A21202) were applied for 2 h at room temperature. Chambers were removed and slides were mounted with mounting Medium with DAPI (Abcam 104,139) and dried overnight. A stitched image corresponding to the area covered by a central 4 × field of view was generated by combining adjacent 40 × images acquired on an Olympus IX83 microscope (UTW0X3 camera, 0.63-inch sensor). The central 4 × window of a well corresponds to an area of approximately 1.28 mm × 0.96 mm and was reconstructed by stitching multiple 40 × images, each covering 0.16 mm × 0.12 mm. All immunofluorescence images were captured using preset microscope settings of 500 ms exposure and gain 5. To assess differences in the expression of cell identity markers between experimental groups, ImageJ (Fiji) was employed to quantify the immunofluorescence-stained area for each marker. The images from each fluorescence channel were converted to 8-bit grayscale to facilitate threshold-based analysis. To distinguish specific marker signals from background fluorescence, threshold levels were adjusted to accurately identify the positive staining area. Following threshold application, the Analyze Particles function was used to measure the fluorescent area corresponding to each marker and plotted relative to total image area. For each sample, the total marker area was additionally normalized to the total cell count area indicated by DAPI.

### 2.7 RNA-sequencing

Total RNA was isolated from untreated, 10 ng/mL hS3 continuously treated, and 20 ng/mL hS3 continuously treated hiPSC- and hESC-derived neuronal cultures on differentiation days 15, 25, and 40 using TRIzol™ Reagent (Thermo Fisher Scientific, 15,596,018). Following DNase digestion with Promega RQ1 RNase-Free DNase (M6101), RNA was purified using a phenol-chloroform extraction protocol, and RNA concentration was measured with a NanoDrop spectrophotometer. All samples passed quality control with an RNA Integrity Number (RIN) higher than 8. Sequencing libraries were prepared according to the NEBNext Ultra II RNA Poly(A) Workflow which included the NEBNext Poly(A) mRNA Magnetic Isolation Module (E7490), NEBNext Ultra II Directional RNA Library Prep Kit for Illumina (E7760L), NEBNext Sample Purification Beads, and NEBNext Multiplex Oligos for Illumina (Set 4, E6443). Each library was generated with an input of 400 ng total RNA per sample. Paired-end RNA libraries were sequenced on the NovaSeq X Plus platform (Illumina) in PE100 mode with a loading concentration of 200 pM. Sequencing was conducted by the Core Facility Genomics at the Berlin Institute of Health (BIH).

### 2.8 RNA-sequencing analysis

Reads were aligned to human genome GRCh3 p7 using the STAR aligner (Dobin et al., 2013), v. 2.7.3a using the GENCODE annotation v. 25. Gene counts were obtained from featureCounts (Liao et al., 2014) from the Subread package, v. 2.0.3. Differential gene expression analysis was performed with the R package DESeq2 (Love et al., 2014), v. 1.38 using the custom seasnap pipeline.^3^ All *p*-values obtained in differential gene expression analysis and gene set enrichment analysis were corrected for false discovery rate (FDR) using the Benjamini-Hochberg procedure (Benjamini and Hochberg, 2018). For the purpose of visualization and principal component analysis, reads were normalized using the regularized logarithm transformation as implemented in the DESeq2 rlog function. We defined differentially expressed genes (DEGs) as genes with FDR < 0.05 and absolute log2 fold change greater than 1 (which corresponds to at least 2-fold change in either direction). For gene set enrichment, we used the CERNO algorithm implemented in the R package tmod v. 0.50.13 (Zyla et al., 2019) combined with gene set definitions from KEGG, Reactome and Gene Ontology as provided by the MSigDB database (Liberzon, 2014) and implemented in the R package msigdbr, v. 7.5.1 (Dolgalev, 2022). As effect size measure, we used the area under curve (AUC) calculated in the tmod package,and considered only at least moderate effect sizes (AUC > 0.65). For DEGs analysis of treated versus untreated we adjusted log2FC threshold to > 0,5 and FDA threshold to < 0,1 to capture subtle but functionally relevant transcriptional changes, particularly for regulatory genes and non-coding RNAs, in datasets with low sample size.

### 2.9 Quantitative real-time PCR (qPCR)

Total RNA was reverse transcribed into cDNA using the SuperScript™ IV Reverse Transcriptase Kit (Thermo Fisher Scientific, Cat# 18090050) according to the manufacturer’s instructions, with random hexamer primers (Thermo Fisher Scientific, Cat# N8080127), 10 mM dNTP mix (Thermo Fisher Scientific, Cat# 18427013), and RNaseOUT™ Recombinant Ribonuclease Inhibitor (Thermo Fisher Scientific, Cat# 10777–019). The reaction was performed using 1 μg of total RNA per 20 μL reaction volume, followed by enzyme inactivation at 80 °C for 10 min. Quantitative PCR was conducted on a QuantStudio™ 6 Flex System (Thermo Fisher Scientific) using TaqMan™ Gene Expression Master Mix (Thermo Fisher Scientific, Cat# 4369016) and TaqMan™ Gene Expression Assays for the respective target genes (ICAM1: Hs00164932_m1; HSPB8: Hs00202470_m1, PGK1: Hs99999906_m1). Reactions were carried out in Fast Thermal Cycling Plates (Thermo Fisher Scientific, Cat# 4346907) with the following cycling conditions: 95 °C –20s, 95 °C –1 s, 60 °C –20s for 45 cycles. Relative gene expression was determined using the ΔΔCt method with PGK1 as housekeeping gene for normalization.

### 2.10 Statistical analysis

Statistical analysis of LDH activity was performed using IBM SPSS Statistics 27. Absolute LDH activity for each well was normalized to the maximum releasable LDH activity of the respective well, yielding the percentage of cell damage. All datasets were tested for normality using the Shapiro–Wilk test and for homogeneity of variances using Levene’s test prior to statistical analysis. Although our study included multiple experimental conditions, statistical comparisons were performed only between each treatment group and its corresponding control, not across all conditions simultaneously. Therefore, the Mann–Whitney U test was applied as the appropriate non-parametric two-group comparison when the data were not normally distributed and/or homogeneity of variances was not met for one of the two conditions being compared. Results are presented as the mean ± standard error of the mean (SEM). A *p*-value < 0.05 was considered statistically significant.

## 3 Results

### 3.1 Detection of EPO and hS3 transcripts in the adult human brain

For co-visualization of EPO and hS3 mRNA in human brain tissue, we utilized the BaseScope Duplex Detection Assay with customized single oligonucleotide probes designed by Advanced Cell Diagnostics to target the specific exon junctions of EPO and hS3 mRNA. According to the BaseScope™ Duplex Detection Reagent Kit User Manual, individual positive signals are visualized as discrete punctate dots corresponding to single RNA molecules. The assay is designed to allow detection of individual target transcripts at 20 × −40 × magnification, where each dot represents a single amplified RNA molecule hybridized by the probe.

**Figure 1:**
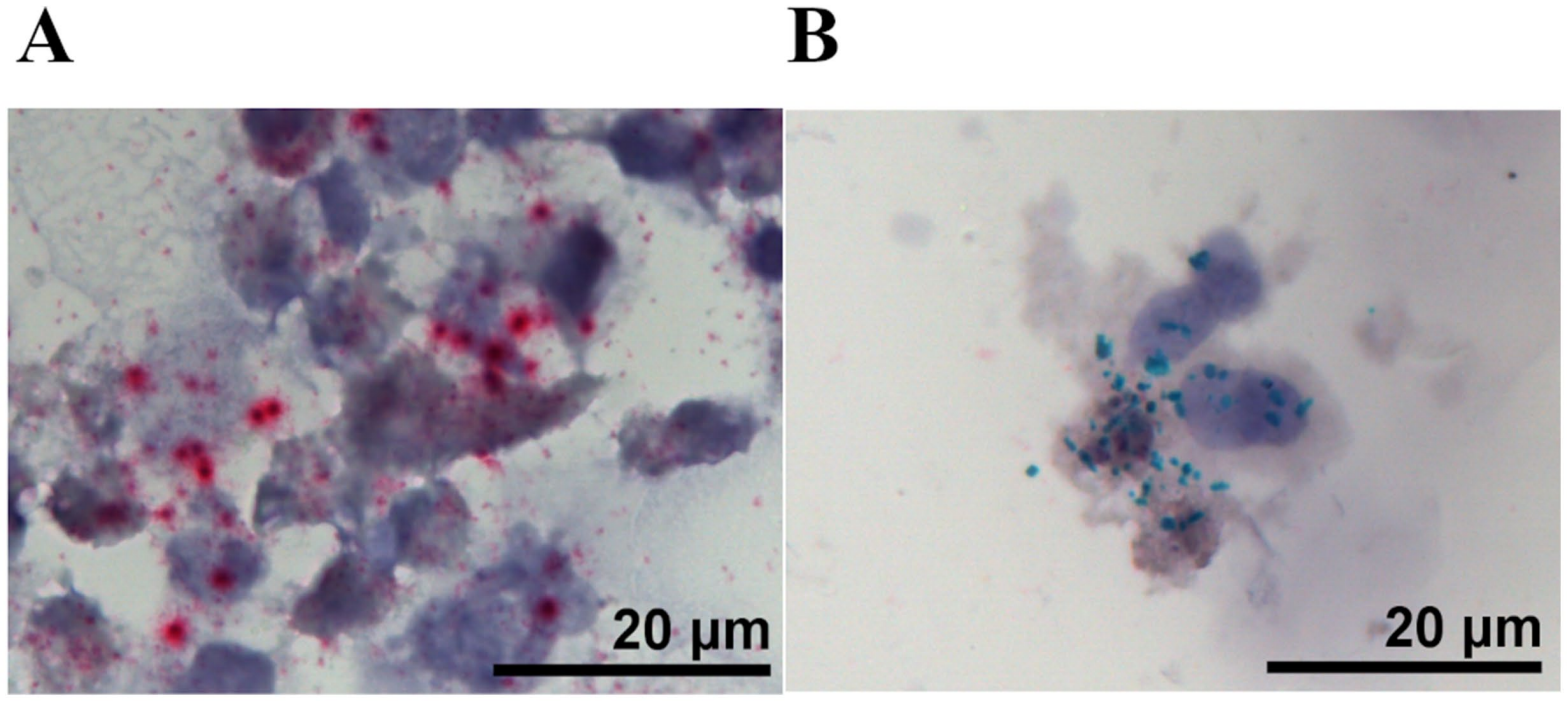
Representative BaseScope duplex assay images showing validation of custom-designed probes in target-expressing CHO cell lines. **(A)** EPO-expressing CHO cells and **(B)** hS3-expressing CHO cells. Both target probes (EPO, red; hS3, green) were applied simultaneously. In EPO-transfected cells, only EPO mRNA was detected, while hS3 signal was absent, and vice versa for hS3-transfected cells, confirming probe specificity. Scale bar: 20 μm.

To verify that the custom-designed EPO and hS3 BaseScope probes specifically detect their intended targets, we first applied both probes to recombinant Chinese Hamster Ovary (CHO) cells expressing either EPO or hS3. The BaseScope staining was performed as a duplex assay, in which both probes (EPO, red; hS3, green) were applied simultaneously to the same section. In EPO-transfected CHO cells, only EPO mRNA was detected, while hS3 signal was absent, and vice versa for hS3-transfected cells, confirming probe specificity without cross-hybridization. This experiment served solely to validate probe functionality (Figure 1).

**Figure 2:**
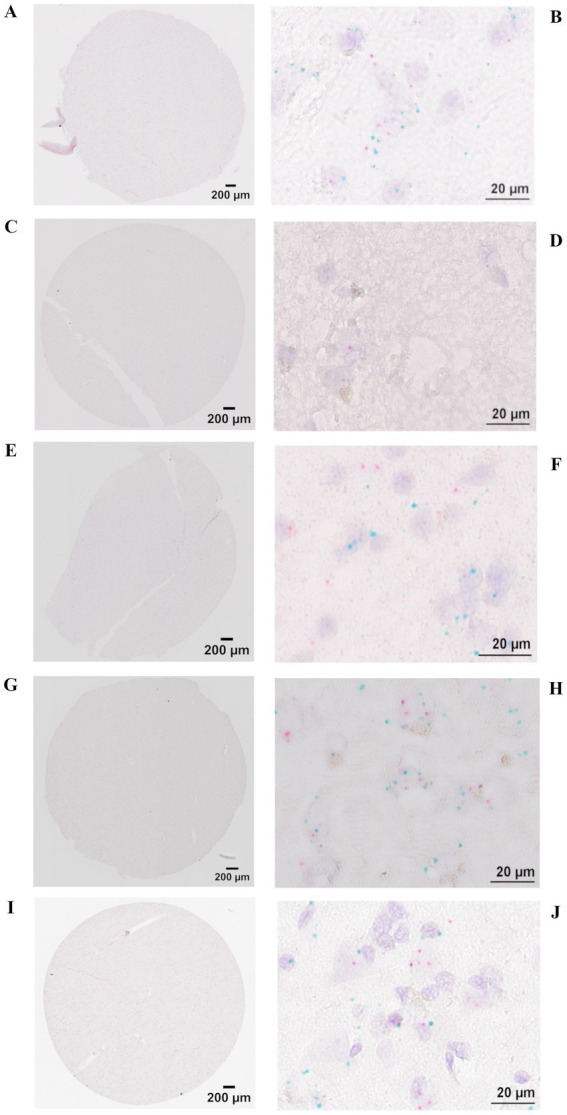
BaseScope representative images of control mRNA stainings for assay validation in individual tissues. Stitched images of 3 mm (area) 5 μm (thickness) sections and image detail taken with a brightfield microscope Olympus IX83 at 40x magnification. Each dot represents one mRNA molecule, red dots represent positive assay control housekeeping gene POLR2A and green dots represent positive assay control housekeeping gene PPIB. **(A,B)** Stroke contralateral hemisphere of ischemic lesion. **(C,D)** Stroke ipsilateral hemisphere of ischemic lesion. **(E,F)** ALS (severe hypoxic encephalopathy after cardiopulmonary resuscitation). **(G,H)** ALS (terminal hypoxia). **(I,J)** PPMS. Detail images show similarly distributed control mRNAs in Stroke contralateral hemisphere of ischemic lesion, ALS (severe hypoxic encephalopathy after cardiopulmonary resuscitation), ALS (terminal hypoxia) and PPMS and reduced positive control mRNAs in necrotic stroke tissue (ipsilateral hemisphere). Scale bar 200 μm stitched image, 20 μm detail image.

**Figure 3:**
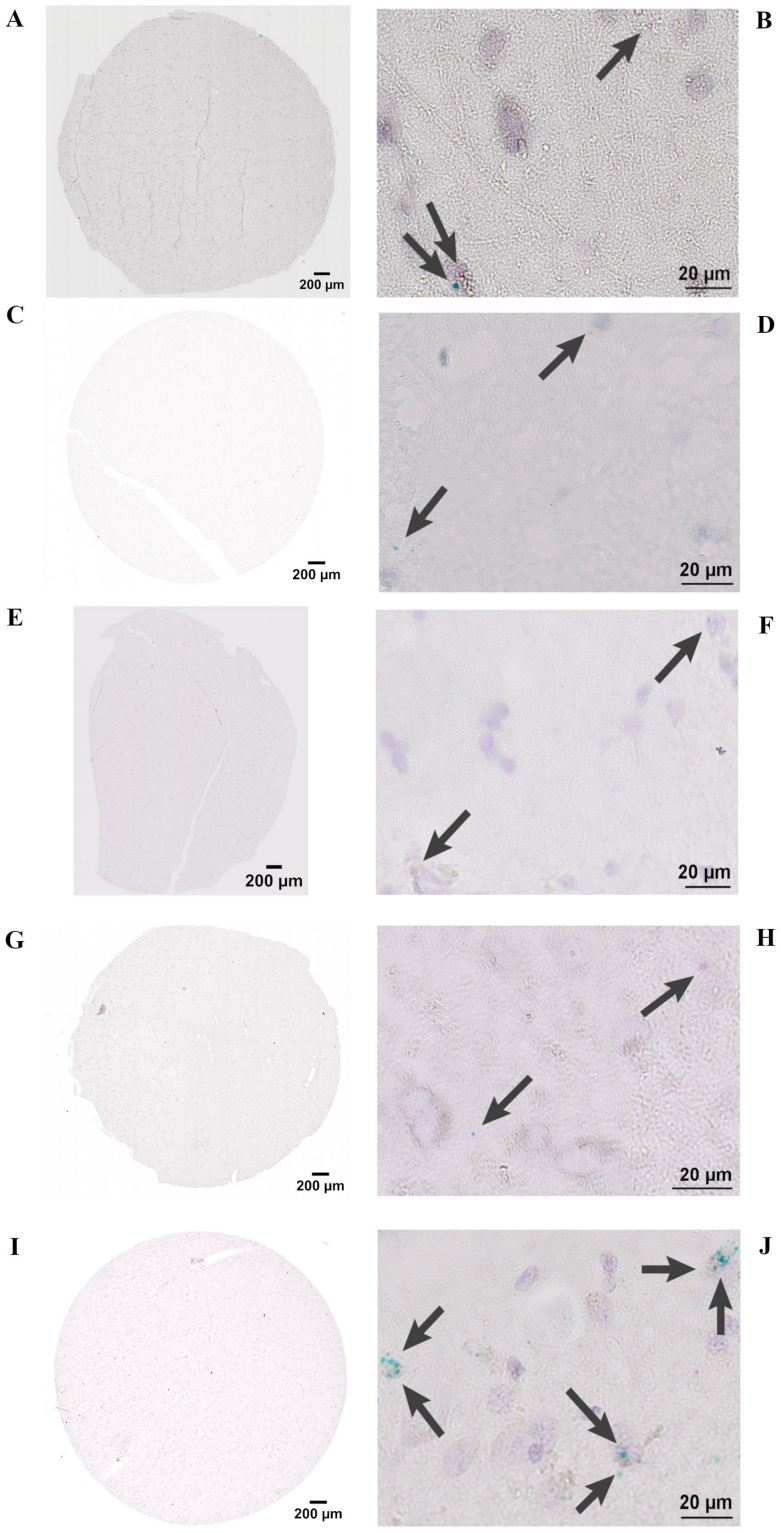
BaseScope representative images of EPO and hS3 mRNA stainings in individual tissues. Stitched images of 3 mm (area) 5 μm (thickness) sections and image detail taken with a brightfield microscope Olympus IX83 at 40x magnification. Each dot represents one mRNA molecule, red dots represent EPO and green dots represent hS3 mRNAs indicated by arrows. **(A,B)** Stroke contralateral hemisphere of ischemic lesion. **(C,D)** Stroke ipsilateral hemisphere of ischemic lesion. **(E,F)** ALS (severe hypoxic encephalopathy after cardiopulmonary resuscitation). **(G,H)** ALS (terminal hypoxia), **(I,J)** PPMS. Scale bar 200 μm stitched image, 20 μm detail image.

To confirm that the BaseScope assay itself performs reliably in different human brain tissues, we applied the manufacturers internal positive control probes to each sample. Positive control probes (PPIB, POLR2A) verify RNA integrity and assay performance in specific human brain samples. These probes serve as assay quality controls rather than normalization references, confirming that the BaseScope assay was functional in the tested tissue. In positive control staining, each red dot represents one mRNA molecule of the housekeeping gene, POLR2A, and each green dot represents one mRNA molecule of housekeeping gene, PPIB (Figure 2). After these verification steps, we applied our custom-designed EPO and hS3 probes to the same human brain tissues to compare transcript expression between samples. In experimental target staining, each red dot represents one EPO mRNA molecule, and each green dot represents one hS3 mRNA molecule (Figure 3).

As expected, the target of interest was expressed only in approximately every tenth to hundredth cell of the brain, depending on disease state, which made a cell-wise scoring (dots per cell) not representative. Therefore, we quantified the total number of mRNA puncta per tissue section for both targets and additionally evaluated the ratio between these two targets detected on the same slide, representing the most meaningful and reproducible way to assess relative expression levels *in situ*. This approach provides a semi-quantitative measure of relative transcript abundance, as it normalizes within-section variability in RNA integrity, probe performance, and amplification efficiency.

**Figure 4:**
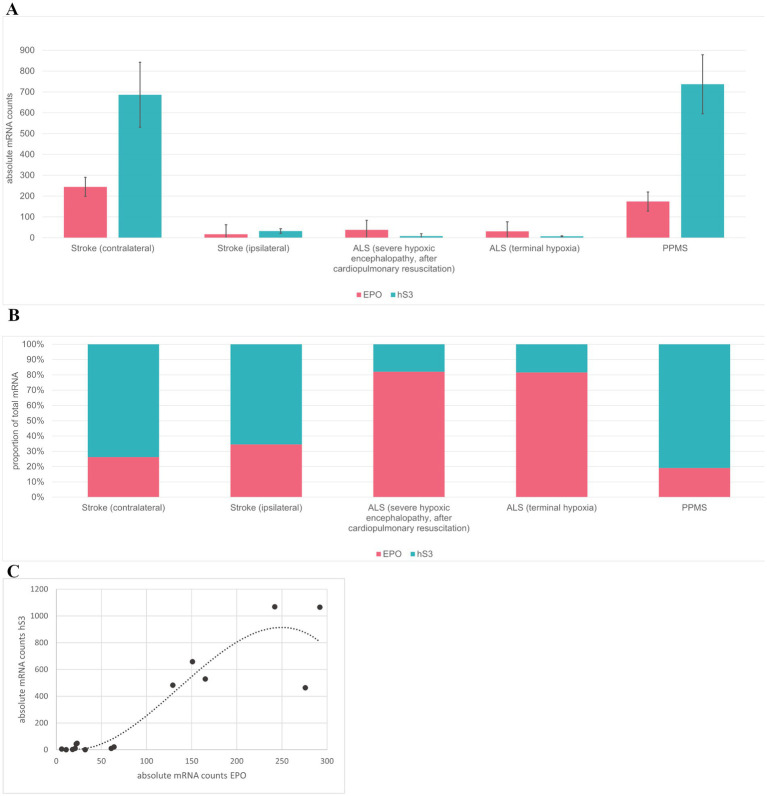
Basescope EPO and hS3 mRNA counts. **(A)** Absolute EPO and hS3 mRNA counts of human brain sections of Stroke contralateral, Stroke ipsilateral, ALS (severe hypoxic encephalopathy, reanimation), ALS (terminal hypoxia) and PPMS. **(B)** Sum of absolute counts were set to 100% to present the EPO/hS3 mRNA ratio. **(C)** EPO and hS3 mRNA co-expression over all tissues and replicates fits a polynomial model of degree 3 y = −0.0002×3 + 0.0618×2–2.3779x + 27.075 R^2^ = 0.8607.

All RNA dots in three sections per brain were manually counted, providing mRNA expression levels for EPO and hS3 (Figure 4A). The lowest hS3 mRNA expression was observed in brain sections from ALS patients, regardless of the severity of hypoxia prior to death. In ischemic brain sections, we detected elevated hS3 mRNA expression, particularly in contralateral regions. The highest hS3 mRNA expression was observed in brain sections from patients with primary progressive multiple sclerosis (PPMS), an inflammatory CNS disease (Figure 4A).

Due to parenchymal destruction within the ischemic stroke brains, absolute mRNA counts obtained using BaseScope may be underestimated and not directly comparable to other tissue samples as demonstrated by inconsistencies of positive control staining (Figure 2D). To account for this, we analyzed the ratio of EPO to hS3 mRNA expression, normalizing the combined counts to 100% (Figure 4B). Assuming that parenchymal destruction affects both EPO and hS3 mRNA counts equally, the EPO to hS3 ratio may provide a more reliable representation of EPO and hS3 mRNA expression dynamics after stroke. In ALS brain sections, EPO mRNA expression exceeded hS3 mRNA expression, while in stroke and PPMS brains, hS3 mRNA expression surpassed EPO mRNA levels.

To explore the relationship between EPO and hS3 co-expression dynamics independent of disease conditions, we plotted the total mRNA counts of both targets from individual brain sections of all included brain tissues and fitted several regression models. The co-expression dynamic between EPO and hS3 mRNAs is best described by a polynomial model of degree 3, yielding the highest R^2^ value of 0.8607 (Figure 4C). This model indicates a non-linear relationship, where an increase in EPO mRNA expression is associated with a disproportionately larger increase in hS3 mRNA expression, suggesting a coupled or co-regulated response under pathological conditions. Due to the overall low expression levels of EPO and hS3 in the human brain, individual cells typically show either a single EPO mRNA or a single hS3 mRNA molecule within a given section. However, some cells display co-expression of both transcripts. For example, Figure 3B shows a cell with co-expression of one EPO and one hS3 mRNA molecule. In brain sections with high hS3 expression, such as those from PPMS cases, cluster-like accumulations of hS3 mRNA molecules can be observed (Figure 3J), potentially resulting from a localized regulatory mechanism. This study provides the first evidence of hS3 mRNA expression in human brain tissue and together, these findings identify hS3 as a previously unrecognized EPO-responsive transcript in the human brain.

We did not quantify control probes primarily because these probes are intended for assay qualification rather than quantitative normalization. First, BaseScope multiplexing is limited to duplex, meaning that control probes cannot be run on the same tissue sections as the two targets. Second, probe chemistries differ between control and target probes, especially with respect to probe length in the case of our very short customized target probes, resulting in intrinsically different detection efficiencies that make their dot counts not directly comparable. Third, so-called housekeeping controls (e.g., PPIB, POLR2A) can be regulated in disease-affected brain tissue, violating the assumption of expression invariance. Nevertheless, their expression may cautiously allow us to place our findings into a broader biological context.

We performed an exemplary quantification of the positive control probes PPIB and POLR2A in brain sections from the contralateral hemisphere of a stroke patient and compared these results with our target probes in sections of the same patient and compared results with expression data from public transcriptomic resources (Human Protein Atlas and GTEx). These datasets report RNASeq values derived from healthy human brain tissue, where EPO is low expressed (≈0.2 nTPM), POLR2A shows low expression (≈0.6 nTPM) and PPIB is highly expressed (≈130–140 nTPM). In our BaseScope analysis, the same relative direction of expression was observed, although the magnitude of difference was smaller (Supplementary Figure 1). This deviation is not unexpected, given that our material does not represent fully healthy tissue and that absolute signal counts obtained by *in situ* hybridization are not directly comparable to nTPM values from bulk RNA-Seq. While stroke, inflammation, and hypoxia are known to broadly affect transcriptional activity, we found no specific reports indicating that PPIB or POLR2A are directly regulated under these conditions in the human brain. The observed stable expression direction therefore supports PPIB and POLR2A as suitable housekeeping genes and the difference in expression magnitude likely reflects methodological and tissue-related variability rather than targeted transcriptional regulation.

### 3.2 Cell-free synthesis of glycosylated hS3

Cell-free synthesis of hS3 yielded consistent protein amounts with sufficient post-translational glycosylation, comparable to the well-established cell-free synthesis of EPO (15 μg/mL) (Supplementary Figures 2A,B). In a proliferation assay with the erythroleukemic cell line, TF-1, commercially available EPO and cell free synthesized EPO (10 ng/mL) strongly induced proliferation. In contrast, non-erythropoietic hS3 did not induce any proliferation (Supplementary Figure 2C).

### 3.3 Establishment of a small-scale OGD and neuroprotection assay in human induced pluripotent stem cell- (hiPSC) and human embryonic stem cell- (hESC) derived neuronal cultures

**Figure 5:**
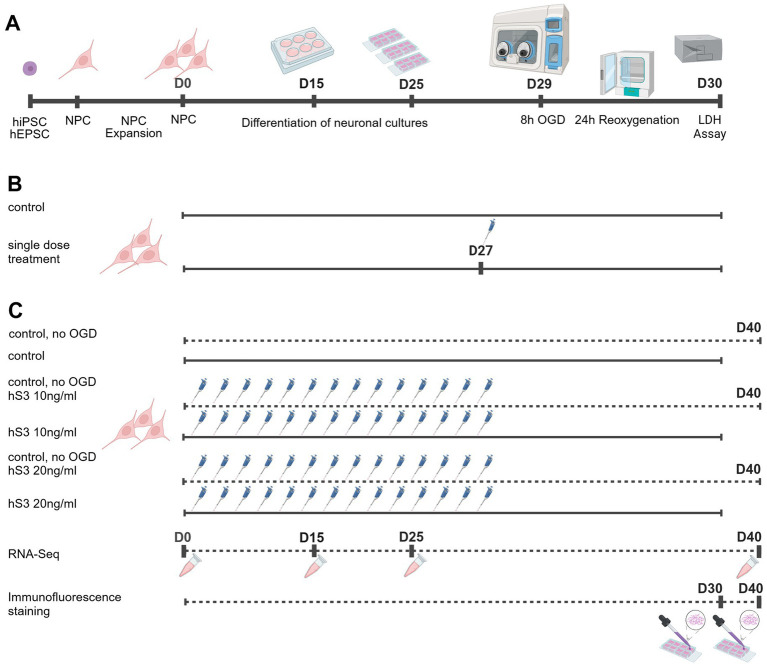
Experimental Design of Cell Culture Experiments. **(A)** Representative timeline of OGD experiments, NPCs were expanded twice before start of differentiation. Cells were replated twice during differentiation. **(B)** Experimental setup of single dose treatment experiments. **(C)** Experimental setup of continuous treatment experiment. For all experiments, neuronal cultures were differentiated from NPCs that originated from a single batch. The NPCs were expanded and passed twice to obtain sufficient numbers, and cells were then evenly distributed into subgroups for parallel differentiation to reduce intergroup variability. Created in https://BioRender.com.

We established a small-scale OGD protocol in human hiPSC-derived and hESC-derived neuronal cultures (Figure 5). The smallest feasible culture setup, limited by evaporation, was achieved using 8-well chamber slides with a working medium volume of 250 μL and a culture area of 0.8 cm^2^ per well, conducted in an Invivo2 400 hypoxic workstation. NPCs derived from hiPSCs and hESCs were seeded at 750,000 cells in 6-well plates, split and replated at the same density on differentiation day 15, and further split and replated at 50,000 cells per well in 8-well chamber slides on day 25. Immunofluorescence staining confirmed the successful differentiation of cells into neurons and glial cells. After 30 days of differentiation, the majority of the cells stained positive for the neuronal marker, class III beta-tubulin (TUBB3, also known as TUJ1), in both cell lines. A smaller subset of cells stained positive for the glial markers, glial fibrillary acidic protein (GFAP) and S100 calcium-binding protein B (S100B). In contrast, no cells showed positive staining for the oligodendrocyte precursor marker, oligodendrocyte transcription factor 2 (OLIG2), or the microglial marker, allograft inflammatory factor 1 (AIF1, also referred to as IBA1) (Supplementary Figure 3A).

For the establishment of the OGD protocol, 5 different OGD durations were tested. The results showed a gradual increase in mean cell damage in untreated hiPSC- and hESC-derived neuronal cultures with increasing OGD duration (Supplementary Figure 3B). For neuroprotection assays, cells were pretreated with 10 ng/mL or 20 ng/mL hS3 for 48 h prior to OGD (Supplementary Figure 3C). Pretreatment concentrations were selected in alignment with previous *in vitro* neuroprotection studies employing EPO or its derivatives (Siren et al., 2001; Bonnas, 2009; Hassouna et al., 2016; Bonnas et al., 2017; Zemella et al., 2018; Knorr et al., 2023). Using the manufacturer’s conversion factor for recombinant human EPO (Roche; 1 IU ≈ 10 ng protein,(Siren et al., 2001)), the concentrations reported in earlier studies correspond to the following approximate mass equivalents: Bonnas (2009) applied 100 U/L (≈ 1 ng/mL), Bonnas et al. (2017) used 3 U/mL (≈ 30 ng/mL), Siren et al. (2001) and Hassouna et al. (2016) each applied 1 U/mL (≈ 10 ng/mL), and Zemella et al. (2018) used 10 and 30 ng/mL in proliferation assays and Knorr et al. demonstrate iPSC-derived neurons being best protected by concentrations of 33,3 ng/mL, or 41,5 ng/mL, respectively. Thus, our chosen concentrations of 10, 20, and 30 ng/mL (≈ 1–3 IU/mL) fall within the range commonly employed in neuroprotection and neuronal differentiation studies, representing a physiologically relevant midpoint for comparison. The 48-h pretreatment was chosen based on prior findings demonstrating that EPO-mediated hypoxic preconditioning requires at least 48 h to establish sufficient ischemic tolerance (Prass et al., 2003). Cell injury was quantified 24 h after reoxygenation using a LDH release assay. To reliably detect neuroprotective effects, a moderate level of injury was required. This was consistently achieved with an OGD duration of 8 h, resulting in approximately 45% cell damage. At higher injury levels (e.g., ~55% after 10 h OGD), neuroprotection was no longer detectable, likely because extensive cell death exceeded the reversible threshold for protective signaling. Based on these results, an 8-h OGD duration was applied in all subsequent experiments. Preliminary results indicated a non-significant trend of greater neuroprotection with a single-dose 48-h pretreatment of 20 ng/mL hS3 compared to 10 ng/mL. The corresponding figure supporting this statement is provided in Supplementary Figure 3C. The experimental design of cell culture studies is depicted in Figure 5.

### 3.4 A single dose of hS3 and hS3-derived peptide protect hiPSC- and hESC-derived neuronal cultures from OGD-induced apoptosis

**Figure 6:**
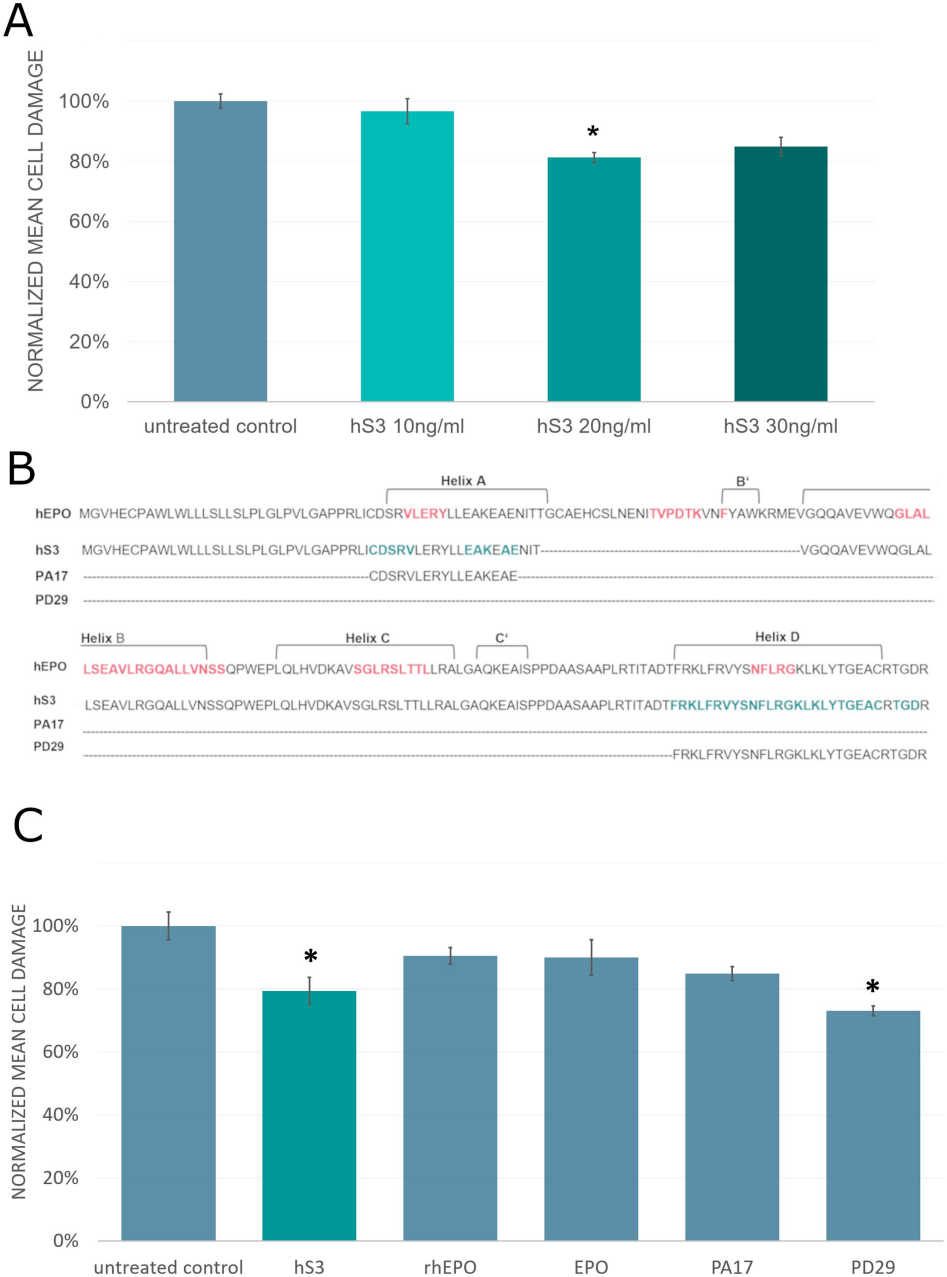
hS3 and D-helix–derived peptide PD29 protect human neurons from ischemic damage. **(A)** Single dose of hS3 protects hiPSC-derived and hESC-derived neuronal cultures from OGD-induced apoptosis. A single dose of 20 ng/mL hS3 at 48 h before OGD significantly reduces cell damage compared to untreated control (*p* = <0.05, Mann–Whitney). Cell damage assessed by LDH Assay of treated cultures was normalized to cell damage of untreated control cultures. Normalized mean cell damage ±SEM is plotted. Untreated control [*n* = 9 independent experiments with 4 replicates (independent wells)], hS3–10 ng/mL (*n* = 7 independent experiment with 4 replicates), hS3–20 ng/mL (*n* = 8 independents experiment with 4 replicates), hS3–30 ng/mL (*n* = 3 independent experiments with 4 replicates). All measurements were performed in duplicates. **(B)** Identification and functional validation of neuroprotective domains in hS3. Protein sequence of human EPO, hS3 and hS3-derived peptides, red indicates EPO binding residues, green indicates precited hS3 binding residues. **(C)** Superior efficacy of the D-Helix–Derived Peptide PD29. Pre-treatments with hS3 and hS3-derived peptide, PD29, significantly reduce cell damage by 21 and 27%, respectively, compared to untreated control (*p* = <0.1, Mann–Whitney). No significant protection was observed for EPO produced by HEK cells (rhEPO), cell free synthesized EPO (EPO) and hS3-derived peptide, PA17. Cell damage assessed by LDH Assay of treated cultures was normalized to cell damage of untreated control cultures. Normalized mean cell damage ±SEM is plotted. Two independent experiments, *n* = 8 replicates (independent wells for each treatment). All measurements were performed in duplicates.

Neuronal cultures were derived from two well-established healthy human reference cell lines, differentiated for 29 days and pre-treated with three different hS3 concentrations (10 ng/mL, 20 ng/mL, and 30 ng/mL) 48 h prior to an 8-h OGD period. Cell damage was assessed 24 h after reoxygenation using a LDH assay. A single-dose of 20 ng/mL hS3 at 48 h prior to OGD significantly reduced cell damage by 18.7% compared to untreated controls (*p* < 0.05, Mann–Whitney) (Figure 6A). Based on these results, subsequent experiments with EPO and hS3-derived peptides were conducted using concentrations of 20 ng/mL.

### 3.5 Identification and functional validation of neuroprotective domains in hS3 reveals superior efficacy of the D-Helix–derived peptide PD29

To compare the neuroprotective effects of cell-free synthesized hS3 with full-length EPO, commercially available recombinant EPO expressed in HEK cells (Sigma-Aldrich E5546) and cell-free synthesized EPO were utilized. Both forms of EPO had previously been validated in TF-1 proliferation assays.

To determine key neuroprotective domains of hS3, we integrated protein modelling and binding site prediction analyses, using various ligand-binding site prediction algorithms (Clark et al., 2020). Template-based methods such as, 3DLigandSite (Wass et al., 2010), Firestar (Lopez et al., 2007), I-TASSER (Zhang, 2008), IntFOLD (Roche et al., 2011), and ProBis (Konc and Janezic, 2010) identify binding sites by referencing structurally similar homologs. Energy-based methods, such as, FTSite (Ngan et al., 2012), predict binding regions by calculating intermolecular interactions like hydrogen bonding and *π*-stacking. Geometry-based methods, including fpocket (Le Guilloux et al., 2009) and dogsitescorer (Volkamer et al., 2012), analyze protein surfaces to identify potential binding pockets based on biophysical parameters such as van der Waals radii. Additionally, a machine learning method (Jendele et al., 2019) integrating various physicochemical properties was employed to improve binding site predictions. Based on these analyses, we identified potentially relevant peptide sequences from the A-helix (PA17) and the D-helix (PD29) of hS3. The amino acid sequences of human EPO, hS3, and hS3-derived peptides are shown in Figure 6B, with EPO binding residues highlighted in red and predicted hS3 binding residues highlighted in green. Peptides were synthesized by Bachem (Bubendorf, Switzerland).

Evidence supporting the biological relevance of the D-helix for neuroprotection was drawn from protein modeling with the SWISS-MODEL web-based integrated protein structure homology modelling service. SWISS-MODEL performs template searches based on structural similarity (Waterhouse et al., 2018) and identified alignment of the C-terminal region containing the D-helix sequence with human caspase-1 and caspase-4, both of which play key roles in inflammatory responses. Notably, the C-terminal sequence of hS3 also aligned with caspase-3 subunit 17, a component of the activated caspase-3 complex, which is essential for apoptosis.

A follow-up experiment was conducted to compare treatments, replicating the protocol used in the previous experiments with hiPSC- and hESC-derived neuronal cultures and different hS3 concentrations. Recombinant EPO expressed in HEK cells (rhEPO), cell-free synthesized EPO (EPO), and the hS3-derived peptide, PA17, showed non-significant reductions in cell damage of 9.6% (rhEPO), 10.0% (EPO), and 15.2% (PA17), respectively, compared to untreated controls. In contrast, treatment with full-length hS3 significantly reduced cell damage by 20.7%, while the novel D-helix-derived peptide, PD29, achieved the highest reduction of 27% compared to untreated controls (*p* < 0.1, Mann–Whitney) (Figure 6C). Additionally, the results demonstrated comparable biological activity between rhEPO and cell-free synthesized EPO, indicating the suitability of cell-free synthesized proteins for iPSC-based models.

Due to differences in molecular weight (EPO ≈ 40 kDa; hS3 ≈ 35 kDa; PD29 ≈ 3.5 kDa; PA17 ≈ 2.0 kDa), the corresponding molar concentrations—and therefore ligand molecule numbers applied—differ particularly between proteins (EPO, hS3) and short peptides (PA17, PD29). Our study therefore compares relative efficacy at equal mass exposure.

### 3.6 Prolonged hS3 administration increases neuroprotection in hiPSC- and hESC-derived neuronal cultures without affecting cell differentiation

**Figure 7:**
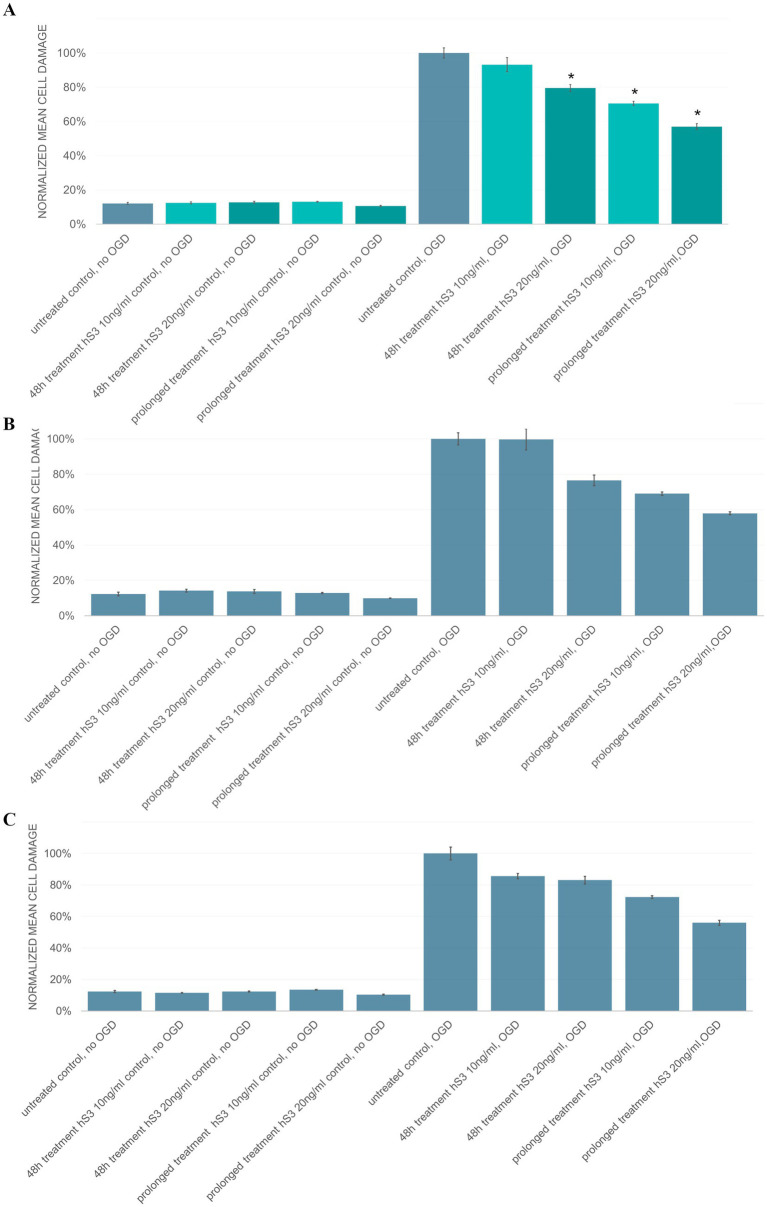
Prolonged hS3 treatment provides neuroprotection in OGD-treated hiPSC-derived and hESC-derived neuronal cultures. Cells were either treated 48 h prior OGD [*n* = 6 independent experiments, 4 replicates (independent wells)], or treated continuously (*n* = 2 independent experiments, 4 replicates) during differentiation starting on day 1 with 10 ng/mL or 20 ng/mL hS3. Normalized mean cell damage ±SEM is plotted. **(A)** Normoxic controls show no difference in cell damage regarding treatment. 48 h treatment with 20 ng/mL hS3 significantly reduces cell damage by 20%, continuous treatment with 10 ng/mL hS3 by 29% and continuous treatment with 20 ng/mL hS3 by 43% compared to untreated control (*p* = <0.05, Mann–Whitney). Effects are equally distributed in **(B)** hiPSC-derived and **(C)** hESC-derived neuronal cultures.

To investigate the effect of hS3 on neuronal development, hiPSC- and hESC-derived NPCs were differentiated in the continuous presence of hS3. Since we could not exclude the possibility that the lower concentration of 10 ng/mL might exert a sufficient or even toxic effect under prolonged exposure, we included both concentrations (10 ng/mL and 20 ng/mL) in these experiments as well. Medium changes were performed every 2 days, with medium supplemented with either 10 ng/mL or 20 ng/mL hS3 starting from day 1 of differentiation. In analogy to the single-dose experiments, OGD was performed on day 29 of differentiation for 8 h, and cell damage was assessed after 24 h of reoxygenation using the LDH assay. Normoxic controls showed no differences in cell damage between hS3-treated and untreated groups, confirming that the observed effects were specific to OGD-induced damage. A single-dose treatment with 20 ng/mL hS3 administered 48 h prior to OGD significantly reduced cell damage by 20.4%, consistent with results from earlier single-dose hS3 experiments (Figure 4C). Continuous treatment with 10 ng/mL hS3 reduced cell damage by 29.4%, while continuous treatment with 20 ng/mL hS3 achieved the highest reduction of 43.0% compared to untreated controls (*p* < 0.05, Mann–Whitney) (Figure 7A). These neuroprotective effects were similar in both hiPSC-derived neuronal cultures (Figure 7B) and hESC-derived neuronal cultures (Figure 7C).

For neural differentiation assays of both hiPSC- and hESC-derived cells, continuously hS3-treated (10 ng/mL and 20 ng/mL) and untreated cultures were maintained in parallel to OGD-treated cultures, with additional cultures differentiated further until day 45. Immunofluorescence stainings for cell identity markers were performed on days 30 and 45, and cells were collected for RNA sequencing at three time points: day 15, day 25, and day 40, from both hiPSC- and hESC-derived neuronal cultures. Both immunofluorescence staining and RNA sequencing yielded consistent results, showing no differences in neural subtypes between hS3-treated and untreated cultures. Immunofluorescence stainings for cell identity markers, including TUBB3, GFAP, S100B, OLIG2, and AIF1, demonstrated comparable cell compositions across groups (Supplementary Figure 4A). The proportion of marker-positive area relative to the total image area was quantified, revealing no statistically significant differences between hS3-treated and untreated cultures for any of the cell markers (Supplementary Figure 4B). Normalization of marker-positive area to the total DAPI-stained cell area also showed no significant differences (Supplementary Figure 4C). These findings were consistent with the expression levels of the corresponding genes as determined by RNA sequencing (Supplementary Figure 4D). Similarly, no differences were observed in the expression of the proliferation marker, MKI67, between hS3-treated and untreated cultures.

According to recent bulk RNA sequencing (RNA-Seq), single-cell RNA sequencing (scRNA-Seq), and single-nuclei RNA sequencing (snRNA-Seq) analyses of the human brain and iPSC-derived neurons (Holst et al., 2019; Burke et al., 2020; Siletti et al., 2023), neuroserpin (SERPINI1) and secretogranin II (SCG2) are primarily considered markers of mature neurons, while aquaporin 4 (AQP4), gap junction protein alpha 1 (GJA1), and GFAP are indicative of mature astrocytes. Similarly, oligodendrocyte transcription factor 1 (OLIG1) and OLIG2 are predominantly markers of oligodendrocyte precursor cells, whereas S100B, myelin basic protein (MBP), and proteolipid protein 1 (PLP1) reflect mature oligodendrocyte identity. Our RNA-Seq data demonstrates a treatment-independent downregulation of oligodendrocyte precursor cell markers, alongside upregulation of mature neuron, mature astrocyte, and mature oligodendrocyte markers in both hiPSC-derived and hESC-derived neuronal cultures. Notably, higher expression levels of the glial markers, GFAP and S100B, in hiPSC-derived neuronal cultures compared to hESC-derived cultures may indicate a potentially superior glial differentiation capacity in hiPSCs or a faster differentiation potential. Furthermore, upregulation of the N-methyl-D-aspartate (NMDA) receptor subunits, GRIN1, GRIN2A and GRIN3A, which are critical for hypoxia-induced neuronal damage, was observed across both cell lines, independent of treatment. This upregulation suggests terminal neuronal differentiation and functional maturation in the cultures (Supplementary Figure 4D).

In order to assess differentiation capacity, we applied neuronal subtype gene sets from Burke et al., derived by deconvolving single-cell RNA-seq datasets of hiPSC-derived neurons as well as human adult and fetal brain samples (Burke et al., 2020). Both hiPSC-derived and hESC-derived neuronal cultures exhibited a significant downregulation of the iPSC gene set and a significant upregulation of adult neuron, astrocyte, and oligodendrocyte gene sets across all three time points compared to the neuroprogenitor state at differentiation day 0 (Supplementary Figure 4E).

### 3.7 Prolonged hS3 treatment results in a dose-dependent differential expression of apoptosis-related protein-coding genes and long non-coding RNAs (lncRNAs) as well as metabolic pathways

The number of significant DEGs increased with higher hS3 concentrations in both cell lines (Supplementary Figure 5A). The expression levels of significantly differentially expressed apoptosis-related genes (Supplementary Figure 5B) and lncRNAs (Supplementary Figure 5C) exhibited a dose-dependent increase. Key differentially expressed apoptosis-related protein-coding genes included tryptophan hydroxylase 1 (TPH1), gamma-glutamyltransferase 5 (GGT5), matrin 3 (MATR3) and leucine rich repeats and IQ motif containing 1 (LRRIQ1). Similarly, significantly altered apoptosis-related lncRNAs included well-characterized genes such as KCNQ1 opposite strand/antisense transcript 1 (KCNQ1OT1), metastasis associated lung adenocarcinoma transcript 1 (MALAT1), miR-17-92a-1 cluster host gene (MIR17HG) and rhabdomyosarcoma 2 associated transcript (RMST). Additionally, several less-characterized apoptosis-related lncRNAs, including FTX:19, ZBED5-AS1, LINC00682, FEZF1-AS1, and ANKRD10-IT1, as well as lncRNAs with unknown functions, such as ENSG00000280128, ENSG00000261572, ENSG00000280439, ENSG00000274422, ENSG00000279759, ENSG00000279425, ENSG00000271895, ENSG00000237781, and ENSG00000278985, were identified as differentially expressed genes. Interestingly, 90.5% of all differentially expressed lncRNAs, including those with unknown functions, showed a dose-dependent upregulation in treated neuronal cultures. The full list of significantly differentially expressed genes in hiPSC- and hESC-derived neuronal cultures treated with 10 ng/mL or 20 ng/mL hS3 is provided in Supplementary Table S1.

Due to limited sample availability, comprehensive experimental validation of the entire RNA-seq dataset could not be performed within the scope of this study. However, two of the differentially expressed genes identified in our dataset, TPH1 and CD34, have previously been reported to be regulated by EPO in other biological contexts, and thereby indirectly validating the robustness of the RNA-seq findings for the splice variant hS3. Specifically, EPO induces TPH1 expression and serotonin synthesis in erythroid progenitors (Sibon et al., 2019), while CD34 is downregulated during EPO-driven erythroid differentiation (Schippel et al., 2025).

For two further genes for which validated primers were readily available, qPCR confirmed the direction of the RNA-Seq changes in with 20 ng/mL prolonged treated neuronal cultures using the same samples (ICAM1: RNA-seq log₂FC − 0.75 vs. qPCR log₂FC − 0.45; HSPB8: RNA-seq log₂FC − 0.06 vs. qPCR log₂FC − 0.10). Differences in effect-size magnitude are expected given the distinct normalization frameworks (DESeq2 vs. single-gene normalization in qPCR).

Gene Set Enrichment Analysis was performed on RNA sequencing data from neuronal cultures continuously treated with 20 ng/mL hS3, using well-defined gene sets from KEGG, Hallmark, and GOBP, as well as metabolic profiling clusters from Weiner et al. (2012). The analysis identified several apoptosis-related metabolic pathways significantly downregulated in both cell lines, including oxidative phosphorylation (OXPHOS), citrate cycle (TCA cycle), mitochondrial electron transport chain (ETC), glycolysis/gluconeogenesis, pentose phosphate pathway, and ribosomal protein production (Supplementary Figure 5D). In hiPSC-derived neuronal cultures, additional hypoxia-relevant gene sets, including reactive oxygen species (ROS) pathway, peptidyl proline hydroxylation, and platelet activation I, were significantly downregulated in response to continuous hS3 treatment. These gene sets were also downregulated in hS3-treated hESC-derived neuronal cultures, although they did not reach statistical significance. A time-point-dependent comparison of continuously treated and untreated neuronal cultures revealed early inhibition of proliferation and cell cycle-related genes on differentiation day 15 in both hiPSC- and hESC-derived neuronal cultures (Supplementary Figure 6). This early inhibition was consistent across both cell lines and suggests that continuous hS3 treatment may promote differentiation in all neuronal subtypes by promoting cell cycle exit.

## 4 Discussion

The non-hematopoietic EPO splice variant, hS3, discovered by us (Meisel et al., 2006) functions as an anti-apoptotic and anti-inflammatory modulator within an endogenous neuroprotective system. Although studies on its biological relevance remain limited, hS3 has demonstrated neuroprotective effects in several models, including hypoxia-induced damage in primary rat hippocampal neurons and rotenone-induced apoptosis in iPSC-derived human neurons (Bonnas, 2009; Bonnas et al., 2017; Knorr et al., 2023). In terms of immunomodulation, hS3 induces the expression of the anti-inflammatory cytokine IL-10, and reduces the expression of pro-inflammatory cytokines, such as IL-6 and IL-8, more effectively than rhEPO in human macrophages stimulated with endotoxin lipopolysaccharide (Meisel et al., 2006). All previously published studies classically apply recombinant proteins produced by transient transfection of CHO cells. To date, no studies have investigated hS3 expression in the adult human brain, nor have they explored its expression in neurological diseases or its role in human neuronal development. Moreover, we are the first to apply stably glycosylated cell-free synthesized proteins in an iPSC-based model, a method that offers a significant advantage for therapeutic development by enabling rapid protein production within hours compared to cell-based systems that require several weeks (Dondapati et al., 2020). Further we identified the D-helix of hS3 as the key protein domain most relevant for neuroprotective effects.

### 4.1 Induction of EPO alternative splicing

Hypoxia and inflammation are known to influence alternative splicing, resulting in altered expression of specific isoforms with significant functional implications (Jakubauskiene and Kanopka, 2021; Marchese et al., 2021). Our results on hS3 expression in the human brain align with Bonnas et al. (2017), demonstrating co-regulation of EPO and hS3. In addition, we show that hS3 mRNA expression exceeds that of EPO under certain conditions.

Because parenchymal destruction in ischemic stroke tissue likely causes underestimation of absolute mRNA counts, we evaluated the EPO to hS3 expression ratio as a more reliable measure of relative expression dynamics. This analysis revealed that hS3 expression exceeded EPO expression in both ipsilateral and contralateral stroke hemispheres and in PPMS brains, whereas EPO predominated in ALS. These findings suggest that hS3 upregulation is particularly associated with hypoxic and inflammatory conditions, while neurodegenerative states such as ALS are characterized by a relative lack of hS3 induction, indicating context-dependent regulation of EPO alternative splicing.

The pronounced upregulation of hS3 mRNA under ischemia- and inflammation-related conditions (e.g., stroke, PPMS) emphasizes the functional importance of EPO alternative splicing in adapting to stress-related functional demands of the cell. Given that both acute and chronic inflammation are integral to the pathophysiology of stroke (Endres et al., 2022), the observed hS3 mRNA upregulation may result from the inflammatory component of stroke pathophysiology. *In vitro* studies have demonstrated that inflammatory stimuli can directly activate HIF-1 signaling in macrophages (Mi et al., 2008) and microglial cells (Oh et al., 2008), subsequently elevating EPO which contributes to recovery (Mengozzi et al., 2008; Luo et al., 2013). However, hypoxia has also been described as immune-independent pathomechanisms in multiple sclerosis (Baecher-Allan et al., 2018), suggesting that both hypoxic and inflammatory pathways may independently regulate EPO alternative splicing into hS3 in the CNS. It is essential to consider that, in addition to splicing-related triggers, various regulatory mechanisms, including thresholds and feedback loops, are likely to contribute to the modulation of hS3 gene expression patterns.

### 4.2 Expression of EPO and splice variant hS3 in the human brain

One of the primary limitations of the BaseScope Duplex Assay is its dependency on tissue quality. To ensure optimal performance, the post-mortem interval should ideally be less than 12 h, and the fixation time between 16 and 32 h. However, these strict criteria exclude many human brain tissue samples. Additionally, since terminal hypoxia is inevitably involved in the process of death, it must be assumed that hypoxia-induced gene expression is partially influenced prior to tissue collection across all samples. These limitations are not exclusive to BaseScope but also apply to other methods, such as qPCR and RNA-Seq in human tissues. Tissue integrity in the stroke region is expected to be compromised compared to the contralateral hemisphere, complicating the interpretation of absolute mRNA counts in affected regions. However, assuming that tissue degradation technically impacts EPO and hS3 mRNA counts alike, we can compare mRNA ratios of brain tissues ipsilateral and contralateral to the ischemic lesion.

Although the majority of hS3 transcripts appear to be localized in neurons, accurate quantification would require a co-staining approach. Attempts to combine BaseScope with either pre- or post-hybridization immunostaining, as described by Fu et al. (2017), were not successful in our hands. More recent spatial transcriptomic approaches, such as Xenium Prime, would in principle be suitable. However, the hS3 sequence does not meet the stringent probe design criteria required for this platform.

This observation is consistent with previous findings demonstrating similar changes in gene expression changes across nearly all cell types in the hemispheres following unilateral middle cerebral artery occlusion (MCAO) in a mouse stroke model (Wu et al., 2023). Furthermore, increased leukocyte recruitment to the contralateral hemisphere has also been observed following stroke, although to a lesser extent than in the ipsilateral hemisphere (Wanrooy et al., 2022). Notably, even neurogenesis is upregulated in the contralateral hemisphere of MCAO-treated mice (Adamczak et al., 2017). Therefore, the contralateral hemisphere in stroke cannot be considered a healthy control. Our study is the first to directly explore EPO and hS3 mRNA expression in human stroke, ALS and PPMS brains. There is one descriptive study which provides evidence that EPO and EPOR are upregulated in human hypoxic brains lacking quantitative validation (Siren et al., 2001), and one study in humans demonstrating serum EPO levels positively correlate with stroke severity and EPO increase with improved functional outcome (Aberg et al., 2001). To date, there are no studies examining EPO expression in the human brain in ALS or PPMS. However, consistent with our findings, EPO levels in the cerebrospinal fluid (CSF) of patients with ALS have been reported to be reduced compared to those in patients with Alzheimer’s disease and individuals with tension-type headache serving as controls. Moreover, CSF EPO levels show a negative correlation with disease progression (Brettschneider et al., 2007). The observed low EPO expression in ALS brains, compared to ischemic and inflammatory conditions, may reflect a dysregulated cytokine pathway as part of the disease pathomechanism, an adaptation to chronic hypoxia, or an abnormal hypoxia response in general (Sato et al., 2012). In contrast, increased immunoreactivity for EPO has been observed in the CNS of SOD1G93A transgenic mice (Chung et al., 2004), but this was limited to less severely affected regions, potentially representing a compensatory upregulation in areas where cytokine signaling pathway remain intact.

Similarly, *in vitro* models of MS, including experimental autoimmune encephalomyelitis and experimental autoimmune neuritis, demonstrate endogenous EPO induction in both the CNS and the peripheral nervous system (Mengozzi et al., 2008; Luo et al., 2013).

Considering that ALS patients experience chronic hypoxia resulting from respiratory insufficiency, it may be appropriate to categorize conditions into acute (stroke) versus chronic hypoxia (ALS), as well as acute inflammation (stroke) versus chronic inflammatory states (PPMS). This differentiation suggests the presence of distinct thresholds or potentially synergistic amplification mechanisms for hypoxia- and inflammation-induced hS3 expression, akin to the regulatory dynamics observed for EPO (Wu et al., 2024).

In this context, the observed cubic polynomial relationship between the expression of EPO and its splice variant across various disease states further supports the notion that this association is not merely dose-dependent. Instead, it may reflect complex regulatory processes shaped by the interplay of neurodegeneration, inflammation, and hypoxia, which differentially affect alternative splicing or mRNA stability.

Although the majority of hS3 transcripts appear to be localized in neurons, accurate quantification would require a co-staining approach. Attempts to combine BaseScope with either pre- or post-hybridization immunostaining, as described by Fu et al. (2017), were not successful in our hands. More recent spatial transcriptomic approaches, such as Xenium Prime, would in principle be suitable for such analyses. However, the hS3 sequence does not meet the stringent probe design criteria required for this platform, currently limiting the ability to precisely resolve its cellular distribution.

Nevertheless, we provide the first demonstration of hS3 mRNA expression in the human brain using BaseScope, an ultra-sensitive *in situ* hybridization technique specifically designed to detect rare, short RNA molecules, particularly to co-detect exon junctions of splice variants at single-cell resolution (Erben et al., 2018; Erben and Buonanno, 2019). As expected, EPO mRNA expression is scarce, showing upregulation in ischemic stroke and downregulation in neurodegenerative diseases. While hS3 follows a similar expression pattern to EPO, it unexpectedly exceeds EPO mRNA expression levels. Notably, we detect the highest mRNA expression levels of EPO and hS3 in PPMS, further emphasizing potential roles in inflammatory CNS diseases.

### 4.3 Establishment of OGD in hiPSC-derived and hESC-derived neuronal cultures

The OGD model is currently the most widely used *in vitro* approach for mimicking stroke, but so far no standardized protocol has been established. Previous studies have predominantly utilized primary neuronal cells from rodents or neuroblastoma cell lines, with OGD durations ranging from 1 to 24 h, with or without reperfusion, and oxygen concentrations varying between 0 and 8%. These variations have resulted in considerable differences in the extent of injury reported across studies (Juntunen et al., 2020). To date, OGD models in iPSC-derived neurons have only been established in larger 24-well plate format (Juntunen et al., 2020; Jiang et al., 2021; Pires Monteiro et al., 2021; Ristola et al., 2021; Rasanen et al., 2022). OGD was either induced by sodium dithionite or cells were incubated in hypoxic conditions with oxygen as low as 1%. An exception involves the incubation of rodent astrocyte co-cultured glutamatergic and GABAergic neurons under anoxic conditions. We are the first to establish a small scale OGD protocol for hiPSC- and hESC-derived neurons at low oxygen of 0.3%, without requiring rodent astrocyte co-culture. According to the literature hiPSC-derived and hESC-derived neurons express NMDA receptors by the second week of differentiation, with hiPSC-derived neurons demonstrating electrical activity within 3 weeks, and hESC-derived neurons exhibiting significant calcium influx by week four (Gupta et al., 2013; Klima et al., 2021; Cheng et al., 2024). This is in accordance with our results showing that NMDA receptor expression is detectable during week two in both hiPSC-derived and hESC-derived neuronal cultures.

### 4.4 hS3-induced neuroprotection

We are the first to use cell-free glycoprotein synthesis to produce a consistently glycosylated, active hS3 for bioactivity studies. Additionally, we establish a miniaturized OGD protocol in hiPSC-derived and hESC-derived neuronal cultures as an in vitro stroke model. In line with previous studies investigating the biological activity of hS3, we confirmed that cell-free synthesized hS3 exhibits non-erythropoietic properties, comparable to cell-based synthesized hS3. Additionally, we demonstrated that cell-free synthesized hS3 provides a comparable extent of neuroprotection in a small-scale OGD model using hiPSC-derived and hESC-derived neuronal cultures. Furthermore, our results confirm the superior neuroprotective effect of a single dose of hS3 compared to EPO, which is in line with the higher expression patterns of hS3 mRNA observed in human stroke brains and supporting the concept of hS3 as a relevant endogenous neuroprotectant. Despite differences in cell types, agent concentrations, and protection assays across studies, the reported extent of neuroprotection induced by a single dose of hS3 consistently ranges between 20 and 30%. These findings are in line with the “sandwich model” concept of threshold damage discussed by Dirnagl and Meisel (2008) and reflect the mean level of neuroprotection (~25%) identified in the largest comparative study of neuroprotective agents in rodent stroke models and clinical trials (O'Collins et al., 2006). The observation that the small peptide, PD29, demonstrates greater efficacy than full-length hS3 remains highly relevant for clinical translation. However, this raises important considerations regarding plasma clearance and immunogenicity, both of which must be addressed in future studies.

While we established a dose–response relationship for hS3 with an optimum at 20 ng/mL, this analysis was not performed for the smaller peptides. Therefore, it remains possible that we did not capture the optimal response range for PD29, whose higher molar concentration at equal mass exposure suggests that maximal efficacy may occur at lower doses. We acknowledge future studies should incorporate equimolar titrations to better assess receptor occupancy and the bell-shaped (optimum-type) dose–response relationships characteristic of EPO-like ligands (Brines and Cerami, 2005).

### 4.5 hS3-induced transcriptional alterations and implications for pathway hypothesis

Administration of EPO results in differentiation of NPCs into neurons in vitro and *in vivo* (Shingo et al., 2001). After traumatic brain injury, EPO restores spatial memory (Lu et al., 2005). Full-length EPO has been shown to promote the differentiation of hippocampal NPCs into pyramidal neurons and oligodendrocytes without affecting proliferation (Hassouna et al., 2016). EPO signaling has been shown to enhance neurogenesis and oligodendrogenesis following spinal cord injury (Zhang et al., 2018). Based on these findings, we hypothesized that hS3 could similarly influence the differentiation of neural progenitor cells. However, our study contradicts this. Given that no differences in cell composition were observed between hS3-treated and untreated cultures, the observed transcriptional changes should be attributed to treatment effects rather than variations in cell types. However, due to the use of bulk RNA sequencing, we are unable to determine in which specific cell types these expression changes occur in response to hS3 treatment. Continuous treatment of neuronal cultures with hS3 resulted in early inhibition of proliferation and downregulation of cell cycle-associated genes compared to untreated controls. Arresting cells in the G1 phase and prolonging the G1 phase has been shown to induce rapid cell differentiation (Ruijtenberg and van den Heuvel, 2016). At later time points, no differences were observed in the expression of cell identity markers or neural subtype-specific gene sets, suggesting that while the timing of differentiation initiation may be accelerated, the capacity for differentiation into all neural subtypes remains unaffected. This observation aligns with the role of CRLF3 as a hS3 receptor, which has been demonstrated to mediate cell cycle arrest at the G0/G1 phase (Yang et al., 2009).

Although still a subject of debate, cell cycle inhibition is increasingly recognized as a potential neuroprotective strategy. Post-mitotic neurons subjected to a mitogenic stimulus such as ROS generated by hypoxia-induced damage can aberrantly re-enter the cell cycle which results in post-mitotic apoptosis (Liu et al., 2010). Thus, cycle inhibition, particularly when it does not impair neurogenesis of neural progenitor cells in the adult brain, may be of significant therapeutic interest.

In healthy neurons, EPO predominantly upregulates genes associated with neurogenesis, synaptic plasticity, and the enhancement of cognitive function (Adamcio et al., 2008; Hassouna et al., 2016). In contrast, in injured neurons, EPO primarily induces the expression of anti-apoptotic genes, including Bcl-xL, IAP2, and XIAP, while concurrently inhibiting the activation of apoptotic mediators, such as caspase-3 and caspase-9 (Sergio and Rolando, 2022). In the present study, we investigated the effects of long-term hS3 treatment on healthy neurons. Transcriptomic analysis of neuronal cultures with continuous hS3 administration (29 days) and untreated controls reveal upregulation of anti-apoptotic and downregulation of pro-apoptotic protein coding genes. Additionally, continuous hS3 treatment induced the upregulation of apoptosis-related LncRNAs and upregulation of LncRNAs with yet unknown function while causing a downregulation of all key metabolic pathways.

The treatment-dependent protein-coding DEGs identified in this study are involved in various apoptosis-related pathways, including the PI3K/Akt pathway, mitochondrial integrity, cytokine signaling, and non-coding RNA regulation (summarized in Supplementary Table S1).

Among the protein-coding DEGs, TPH1 and CD34 showed expression changes that may have additional biological relevance beyond apoptosis-related signaling. While hS3-associated TPH1 up-regulation and CD34 down-regulation may indicate enhanced erythroid maturation and local survival signaling comparable to EPO, these changes do not per se entail an increase in red cell mass or thrombosis risk; such outcomes typically require sustained systemic drivers of erythropoiesis (e.g., prolonged high EPO exposure) and/or platelet hyperreactivity. As our study does not include a condition with prolonged EPO treatment, the relative magnitude of these effects under hS3 stimulation cannot be determined. Whether and how hS3 may influence coagulation remains unknown; to date, only its lack of erythropoiesis-stimulating activity, in contrast to EPO, has been demonstrated. The potential impact of hS3 on hemostasis will therefore be addressed in future studies. Furthermore, hS3-associated TPH1 up-regulation in neurons could enhance local serotonergic tone, supporting neuroprotection and synaptic plasticity rather than proliferation, as serotonin can suppress microglial activation and promote BDNF release. This interpretation may be further supported by hS3-associated CD34 down-regulation in neural tissue, suggesting normalization of reactive angiogenesis and stabilization of the neurovascular microenvironment.

Accumulating evidence shows that LncRNAs play a critical role in the pathogenesis of neurological disease, such as ischemic stroke and multiple sclerosis, by regulating apoptosis and inflammation (Ghaderian et al., 2020; Wang et al., 2022; Xu and Zhang, 2022) and are considered potential prognostic biomarkers and targets for novel therapeutic approaches. It is particularly noteworthy that LncRNA KCNQ1OT1, MALAT1, MIR17HG, and RMST are upregulated in hS3-treated healthy neuronal cultures, as these lncRNAs are known to be highly upregulated in stroke and inflammatory diseases (Wang et al., 2022). Among them, MALAT1, has been extensively studied for its role in ischemic stroke and MS pathogenesis, where it decreases OGD-induced apoptosis (Khoshnam et al., 2024) and regulates Th1/Th2 cell imbalances (Mohan et al., 2024). Similarly, MIR17HG, the host gene of the MIR17-92 cluster, promotes NPC survival after stroke (Liu et al., 2013) and plays a critical role in Treg development in EAE (de Kouchkovsky et al., 2013). However, some studies suggest pro-apoptotic and pro-inflammatory effects of MALAT1 and MIR17HG, particularly under pathological conditions (Zhang et al., 2022; Khoshnam et al., 2024). Correspondingly, KCNQ1OT1 and RMST have been reported to promote OGD-induced apoptosis and inflammation (Cheng et al., 2020; Ren et al., 2020; Wang et al., 2020).

Additionally, our results suggest that hS3 alters mitochondrial function, leading to further modulation of downstream signaling pathways that culminate in anti-apoptotic, anti-inflammatory and anti-proliferative effects. The observed downregulation of key metabolic pathways, such as OXPHOS, TCA cycle, ETC, Glycolysis/Gluconeogenesis, Pentose Phosphate Pathway and Ribosomal Protein Production, has gained recognition as an effective neuroprotective strategy (Stenzel-Poore et al., 2003; Yenari et al., 2008). This metabolic downregulation represents an adaptive mechanism that enables neurons to survive under stress by reducing ROS production, thereby limiting oxidative damage, conserving energy for essential survival processes, and minimizing metabolic overload to maintain mitochondrial integrity. These changes are critical for improving neuronal survival by enhancing antioxidant capacity and preventing mitochondrial dysfunction. These concepts, along with immunosuppression and hypocoagulation, are well-established mechanisms underlying hypoxic preconditioning and hibernation (Stenzel-Poore et al., 2003; Stenzel-Poore et al., 2004; Dirnagl and Meisel, 2008). Notably, while individual studies have explored the modulation of specific metabolic pathways in relation to neuroprotection, the simultaneous downregulation of all these key pathways in human neuronal cultures has not been previously reported. The present study demonstrates that hS3 is an effective metabolic reprogramming agent, partially mimicking pathways which are relevant for hypoxic preconditioning observed in ischemic injury. However, a comprehensive understanding of the collective impact of these metabolic changes on neuronal survival requires further investigation.

While these transcriptomic findings provide novel insights into the cellular response to hS3, certain methodological limitations should be acknowledged. A limitation of this study is the lack of independent comprehensive experimental validation of the RNA-seq results, which was not feasible due to limited sample material. Future work will aim to validate key differentially expressed genes using complementary experimental approaches.

The neuroprotective EPO receptor, CRLF3, initially identified in insects, has recently been confirmed as the neuroprotective receptor for hS3 in humans (Knorr et al., 2023). Protein modeling and sequence prediction analysis of hS3 indicates the A-helix and D-helix as potential binding sites critical for bioactivity. Application of a novel D-helix-derived small peptide, PD29, which has higher neuroprotective capacity than full-length EPO, full-length hS3, and A-helix-derived peptides, suggests that the D-helix likely contains primary CRLF3 binding sites.

It can be hypothesized that the distinct biological functions of CRLF3 are, at least in part, mediated by hS3. The presence of an increased ligand does not necessarily produce the same outcomes as increased receptor expression, and vice versa, yet CRLF3 knockout studies could be interpreted as models of hS3 deficiency. CRLF3 is an evolutionarily conserved member of the class I cytokine receptor family which has been implicated in diverse biological processes including thrombopoiesis, immune regulation, neuronal development and neuroprotection, and has been associated with a variety of diseases including neurofibromatosis type I, autism spectrum disorders and ALS (Yang et al., 2009; Cirulli et al., 2015; Wegscheid et al., 2021; Bennett et al., 2022; Knorr et al., 2023; Wilson et al., 2023). CRLF3 overexpression induces cell cycle arrest in the G0/G1 phase, while inhibition promotes an increase in the S-phase (Yang et al., 2009). CRLF3 deficiency in mice leads to a sustained thrombocytopenia (25–48%) (Bennett et al., 2022). CRLF3 is involved in adaptive immune response (Polewko-Klim et al., 2020), regulates neurogenesis, neuronal morphology, dendritic development and biogenesis and transport of synaptic vesicles (Hashimoto et al., 2012) but has no effect on neural stem cell proliferation (Wegscheid et al., 2021). Consistent with our findings, CRLF3 mutant mice brains show no differences in the composition of astrocytes, microglia, or oligodendrocytes. Additionally, single-cell RNA sequencing of CRLF3 mutant and wild-type hippocampi revealed no differences in neuronal subpopulations, further supporting the notion that CRLF3 deficiency does not alter neuronal subtype composition (Wilson et al., 2023). These findings would align with our results demonstrating that hS3 influences neuronal development by enhancing apoptotic resilience without promoting proliferation or affecting neuronal subtype differentiation.

It is well-established that cytokines commonly share signal transmitting receptors (Boulanger and Garcia, 2004) and that receptor recognition sites of cytokines are organized as modules that are exchangeable even between cytokines with sequence identity as little as 6% (Sato et al., 2012). According to SWISS-MODEL predictions, hS3 and the caspase-3 subunit 17 share 23.7% sequence identity, which initially led us to consider that the C-terminal sequence of hS3, particularly the PD29 peptide, might structurally mimic caspase-3 subunits and potentially act as a pseudo-substrate, forming inactive caspase-like complexes. Similar pseudo-caspase behavior has been described for the human FLICE-like inhibitory protein (c-FLIP), the only bona fide pseudo-caspase identified so far (Smyth et al., 2020).

However, there is currently no experimental evidence that hS3 enters the cytosol or directly associates with caspase-3. The loss of hS3-mediated neuroprotection in CRLF3-knockout neurons (Knorr et al., 2023) strongly supports membrane-initiated, CRLF3-dependent signaling as the principal mechanism of anti-apoptotic action. Both our study and that of Knorr et al. applied extracellular hS3, not intracellularly overexpressed hS3; thus, if any intracellular effect existed, it would more likely arise from intracellular retention or receptor-mediated internalization rather than spontaneous cytosolic entry. We therefore regard the notion of intracellular mimicry as speculative but testable, pending evidence from biochemical or trafficking studies.

Importantly, this framework also clarifies that a direct, CRLF3-independent inhibition of caspase-3 by hS3 would mechanistically contradict the CRLF3-dependent indirect suppression of caspase-3 expression described by Knorr et al. Nevertheless, these two mechanisms—direct inhibition of caspase-3 activity and indirect transcriptional suppression through CRLF3 signaling—could theoretically coexist and contribute additively to the overall neuroprotective effect of hS3.

Finally, the elevated cleaved-caspase-3 immunoreactivity observed in hS3-treated CRLF3-KO neurons (Knorr et al., 2023) might be reconciled with this model if the antibody also detects inactive caspase-like complexes, which remain immunoreactive but are functionally inhibited.

Although we cannot exclude an intracellular contribution of hS3, there is presently no evidence for cytosolic retention or direct interaction with caspase-3; the available data instead support CRLF3-mediated, membrane-proximal signaling that secondarily limits caspase-3 activation.

Future studies should clarify how hS3 mediates its anti-apoptotic, anti-inflammatory, and anti-proliferative effects via CRLF3, and whether it additionally forms pseudo-caspase-like complexes, interacts with alternative receptors, or engages yet-unidentified signaling pathways.

In summary, we demonstrate that hS3 is expressed in the human brain across ischemic and inflammatory conditions, encompassing both acute and chronic neurological states. Our findings suggest that hS3 functions as an anti-apoptotic and anti-proliferative agent, promoting transcriptomic and metabolic reprogramming that enhances resilience to ischemic injury in hiPSC and hESC-derived neuronal cultures. We identify the D-helix as a peptide sequence conferring the highest degree of neuroprotection. Further studies with larger sample size and the application of iPSC-derived disease models will be required to validate our findings and explore the therapeutic potential of hS3 and its derivative small peptide, PD29, in hypoxia- and inflammation-related neurological disease.

## Data Availability

The RNA-sequencing data for this study have been submitted to the Gene Expression Omnibus (GEO) repository under accession number GSE314054 and can be accessed via the following link: https://www.ncbi.nlm.nih.gov/geo/query/acc.cgi?acc=GSE314054. Further raw data supporting the conclusions of this article will be made available by the authors upon reasonable request directed to the corresponding author.

## Footnotes

1
https://hpscreg.eu/cell-line/BIHi005-A

2
https://www.wicell.org/home/stem-cells/catalog-of-stem-cell-lines/wa01.cmsx

3
https://github.com/bihealth/seasnap-pipeline

## Glossary

EPO: erythropoietin
CNS: central nervous system
HIF-1: hypoxia-inducible factor 1
Treg: regulatory T cells
MS: multiple sclerosis
EPOR: homodimeric EPO receptor
βCR: β common receptor
EphB4: ephrin receptor B4
CRLF3: cytokine receptor-like factor 3
EAE: autoimmune encephalomyelitis
ALS: amyotrophic lateral sclerosis
BBB: blood–brain barrier
HBP/ARA290: helix B surface peptide
CHBP: cyclic helix B peptide
FFPE: formalin-fixed paraffin-embedded
BLAST: Basic Local Alignment Search Tool
NPC: neural progenitor cell
OGD: oxygen glucose deprivation
hiPSC: human induced pluripotent stem cells
hESC: human embryonic stem cells
lncRNAs: long non-coding RNAs
LDH: lactate dehydrogenase
DEG: differentially expressed gene
PPMS: primary progressive multiple sclerosis
KCNQ1OT1: KCNQ1 opposite strand/antisense transcript 1
MALAT1: metastasis associated lung adenocarcinoma transcript 1
MIR17HG: miR-17-92a-1 cluster host gene
RMST: rhabdomyosarcoma 2 associated transcript
ROS: reactive oxygen species
CSF: cerebrospinal fluid
