# Supplementary material for: Non-hematopoietic erythropoietin splice variant is produced in the diseased human brain and confers neuroprotection
Source: Front Cell Neurosci. 2026 Jan 12;19:1677505. doi: 10.3389/fncel.2025.1677505 (PMC12832296; doi:10.3389/fncel.2025.1677505)
Supplement: Supplementary file 2 [file Data_Sheet_1.pdf]

Supplementary Material

Supplementary Figure 1

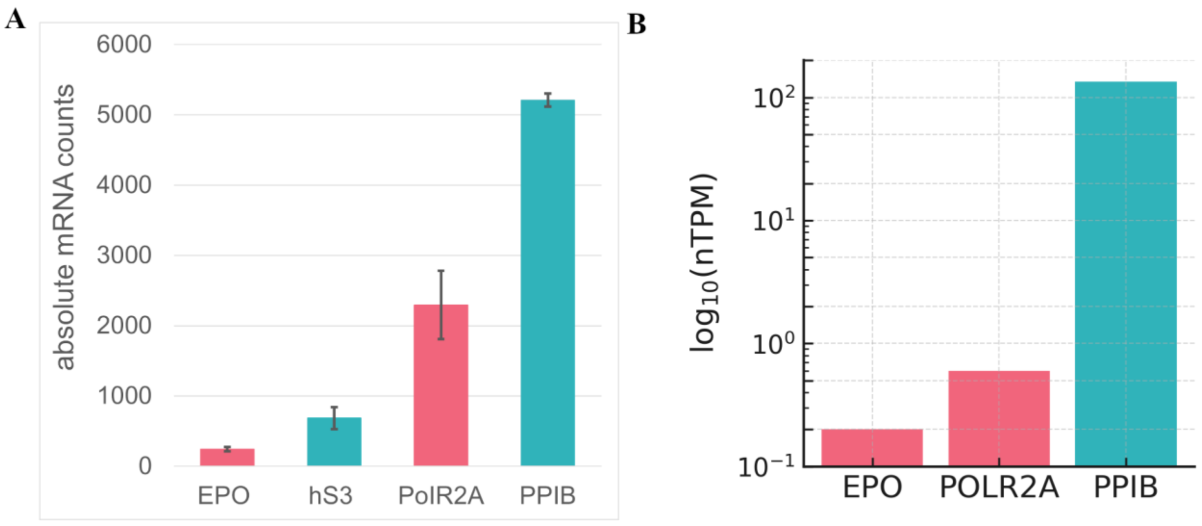

Supplementary Figure 1. Illustrative example of BaseScope target (EPO, hS3) and positive-control (POLR2A, PPIB) mRNA counts in sections of the contralateral hemisphere of a stroke patient and public RNA-seq data from healthy brain tissue. (A) Absolute EPO, hS3, POLR2A, and PPIB mRNA counts in sections of the stroke contralateral hemisphere. (B) External orientation from healthy-brain RNA-seq (HPA/GTEx) data. Transcript abundance (nTPM) is shown on a $\log_{10}$ scale to visualize the large dynamic range, EPO 0,2≈ 0.2 nTPM, POLR2A ≈ 0.6 nTPM, PPIB ≈ 130–140 nTPM. The preserved direction (PPIB > POLR2A) in BaseScope supports assay validity, whereas differences in magnitude likely reflect methodological and tissue variability.

Supplementary Figure 2

A

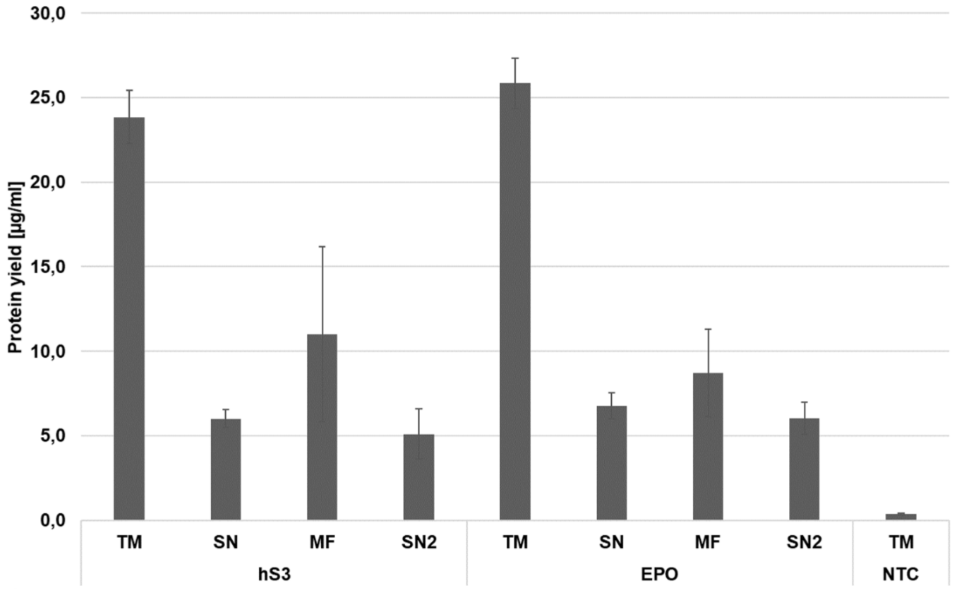

B

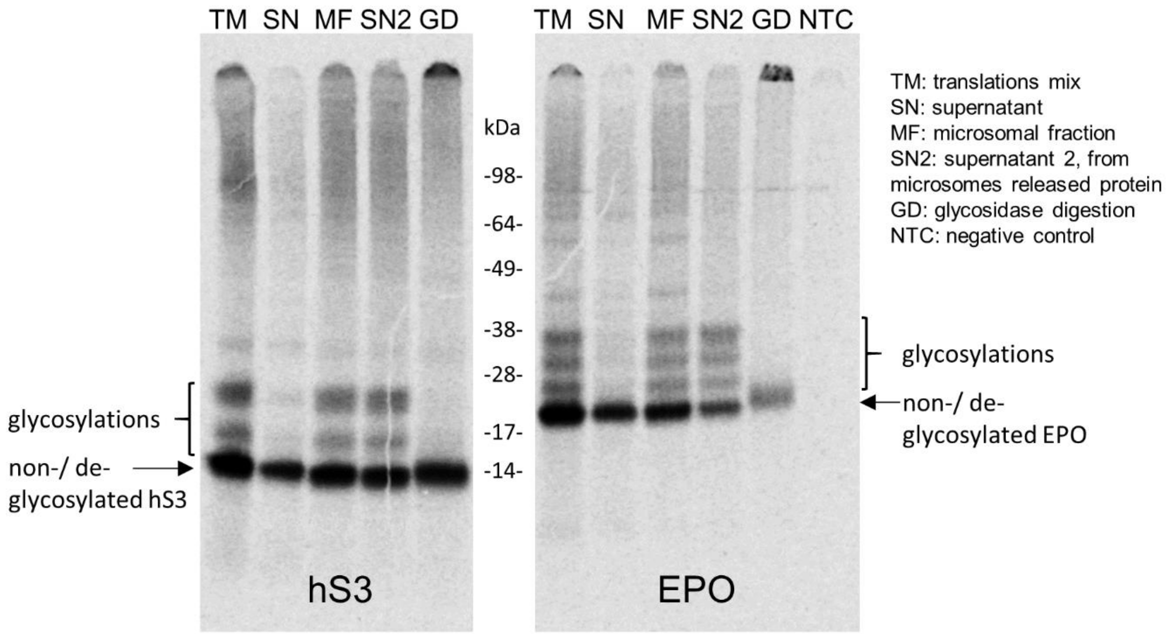

C

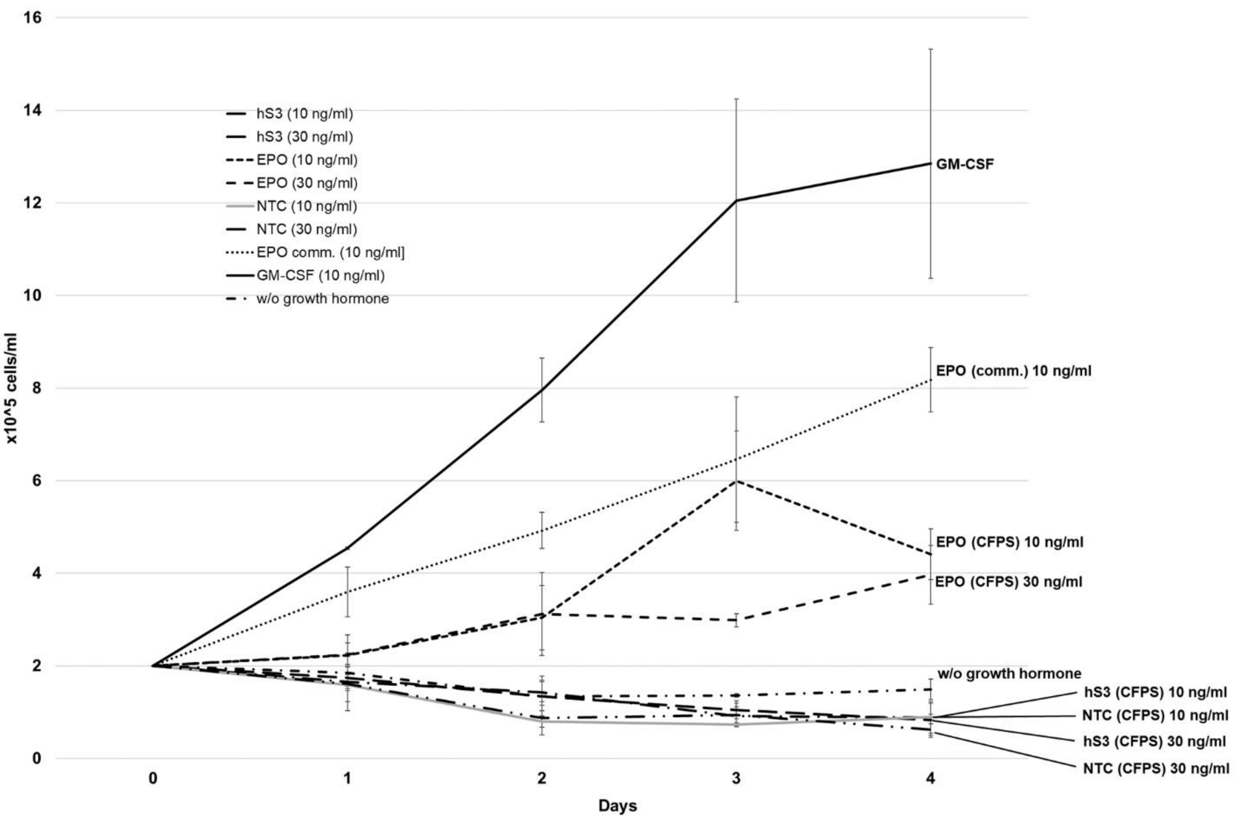

Supplementary Figure 2. Cell-free synthesis of glycosylated hS3 and EPO. **(A)** Quantitative analysis of synthesized hS3 and EPO by liquid scintillation counting. For both proteins comparable yields were determined. **(B)** Qualitative analysis of synthesized hS3 and EPO by autoradiography. A defined band pattern was detected for both proteins including N-glycosylation. **(C)** Cell culture-based proliferation assay. hS3 and EPO were added to a growth hormone dependent cell line. Proliferation was observed after the addition of cell-free synthesized EPO, but not after the addition of hS3.

Supplementary Figure 3

A

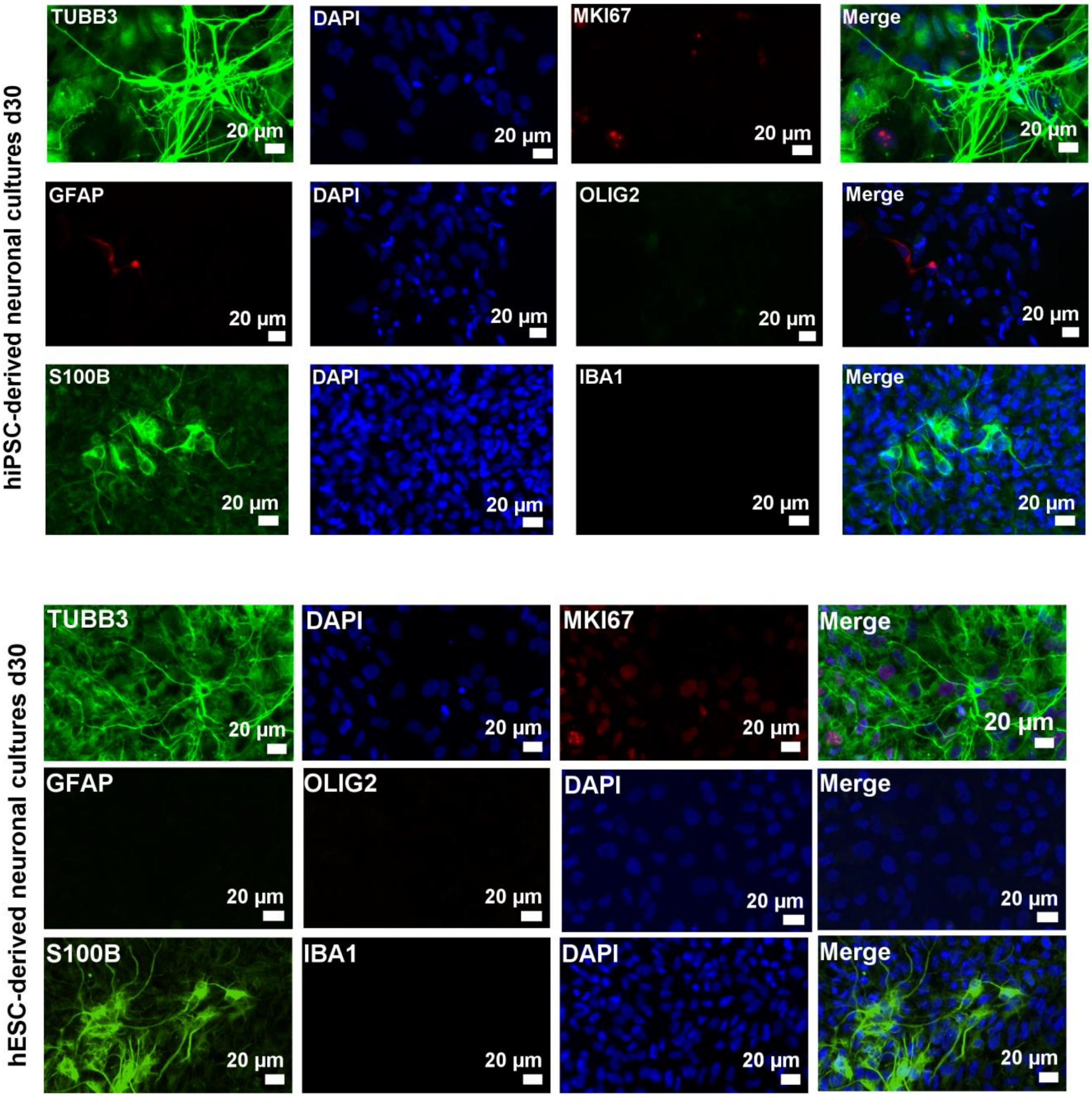

B

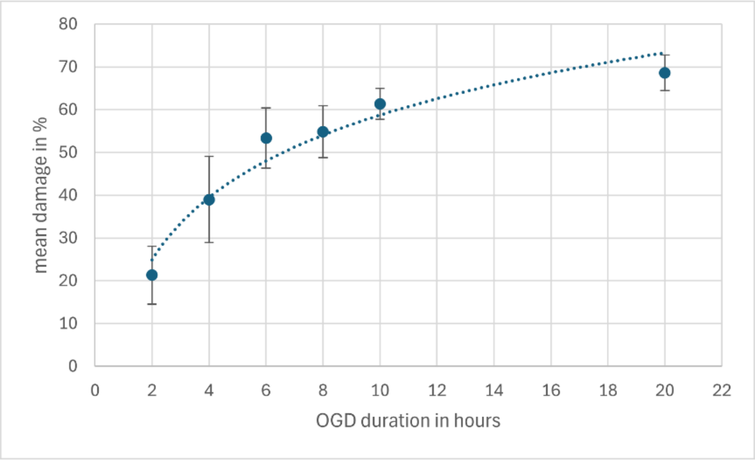

C

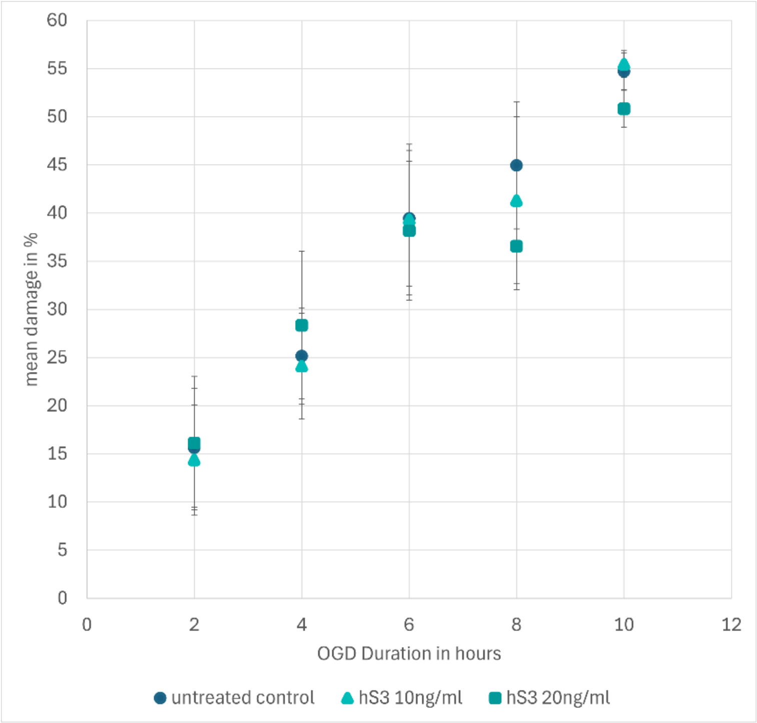

Supplementary Figure 3. Establishment of a small-scale OGD and neuroprotection assay **(A)** Immunofluorescence staining of cell identity markers in hiPSC-derived and hESC-derived neuronal cultures differentiation day 30, Scalebars =20µm **(B)** Mean ±SEM cell damage assessed by LDH Assay of untreated control hiPSC and hESC derived neuronal cultures increases logarithmically with increasing OGD duration. $y = 21{,}013\ln(x) + 10{,}355$ $R^2 = 0{,}9508$ n = 8 independent experiments with 2 replicates **(C)** Mean ±SEM cell damage assessed by LDH Assay of untreated and treated hiPSC and hESC derived neuronal cultures. A minimum of 45% cell damage is necessary to sufficiently measure neuroprotection and is achieved by OGD duration of 8h. A single dose pretreatment 48h prior OGD of 20ng/ml shows a nonsignificant trend of higher protection than 10ng/ml n = 4 independent experiments with 2 replicates

Supplementary Figure 4

A

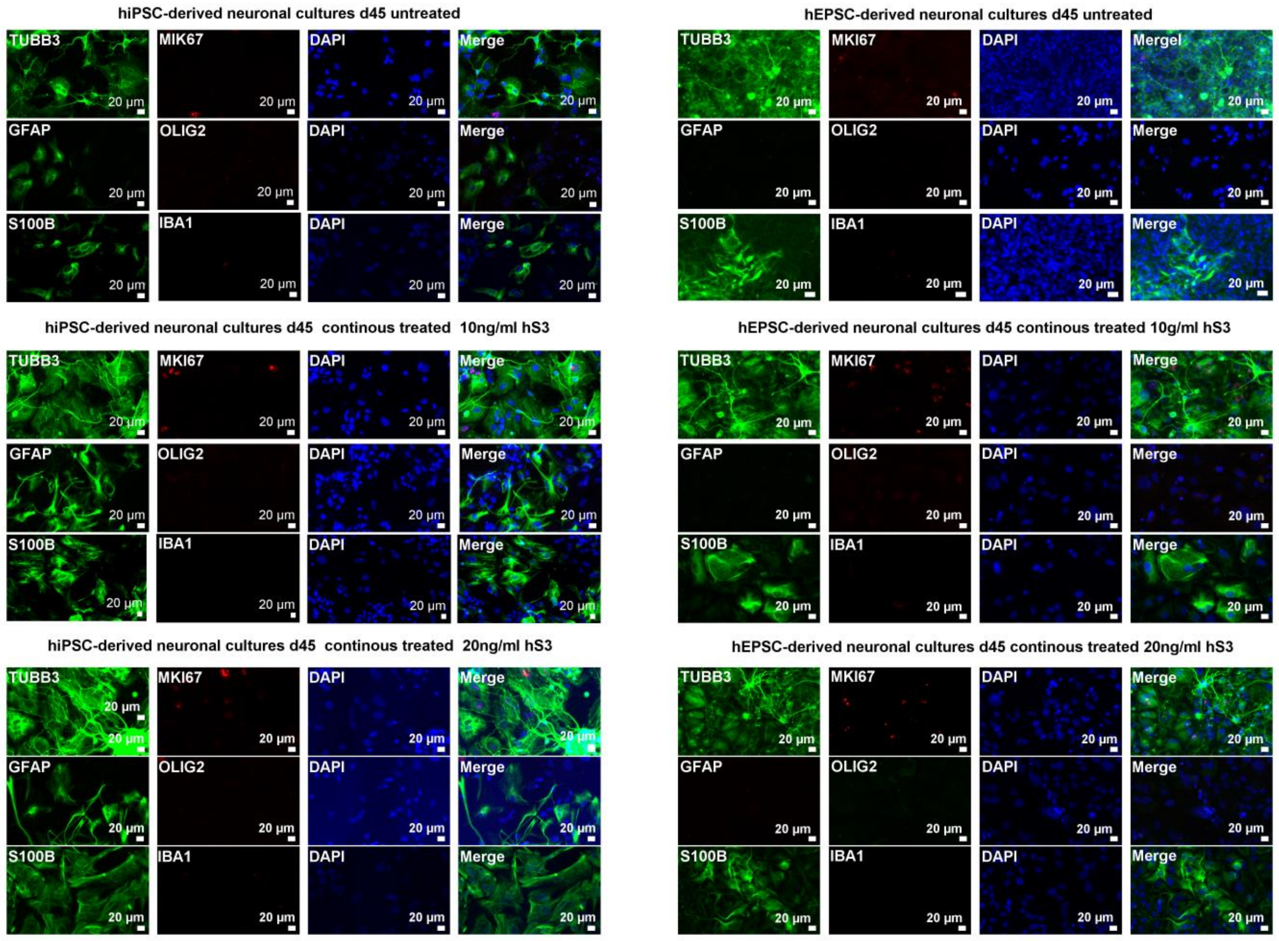

B

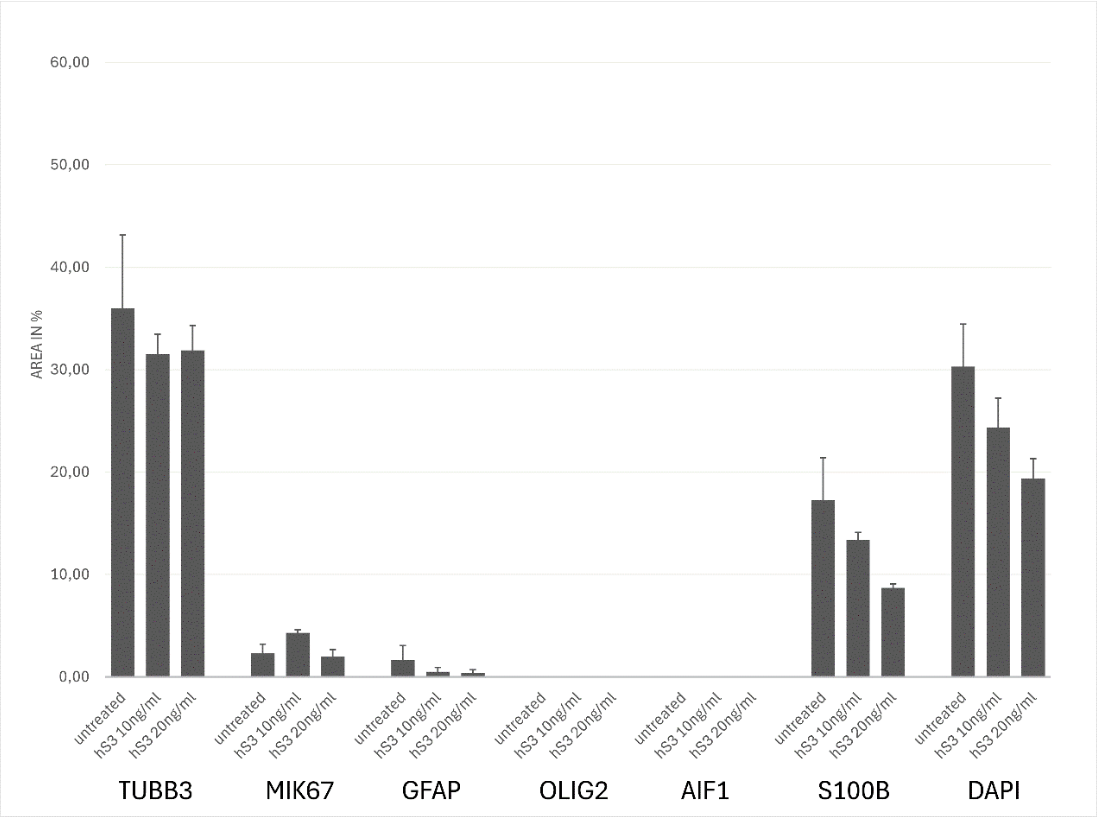
60,00
50,00
40,00
30,00
20,00
10,00
0,00
AREA IN %
untreated
hS3 10ng/ml
hS3 20ng/ml
TUBB3
MIK67
GFAP
OLIG2
AIF1
S100B
DAPI

C

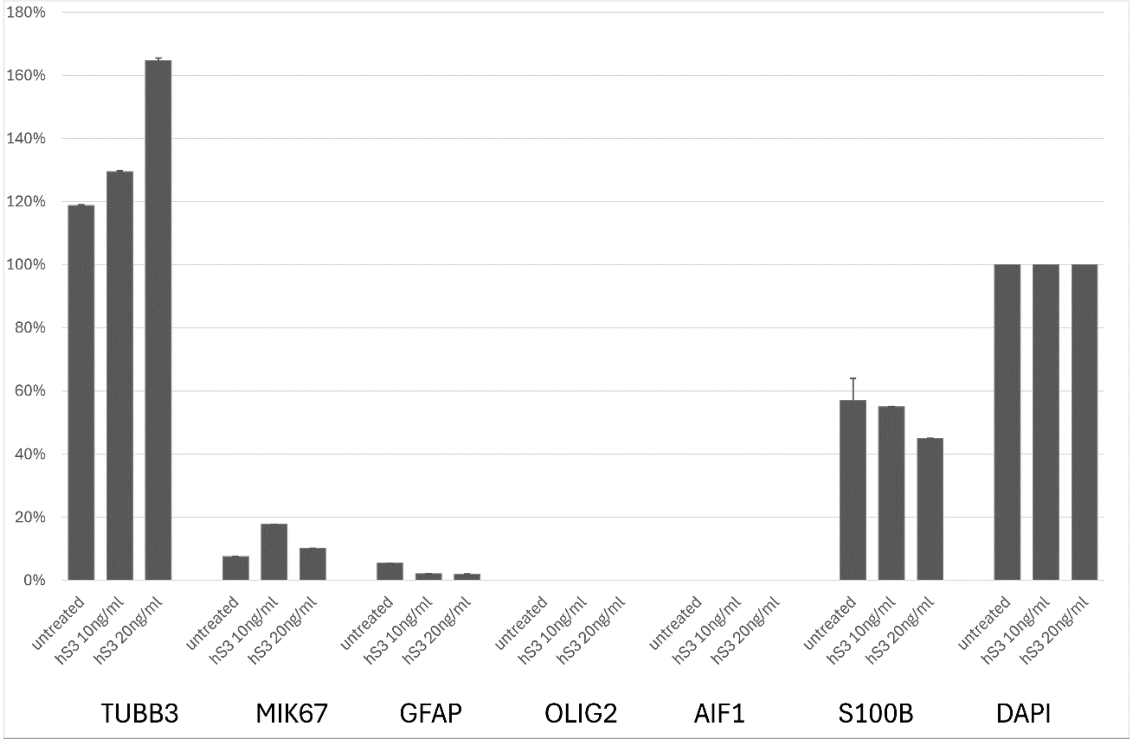
180%
160%
140%
120%
100%
80%
60%
40%
20%
0%
untreated
hS3 10ng/ml
hS3 20ng/ml
TUBB3
MIK67
GFAP
OLIG2
AIF1
S100B
DAPI

D

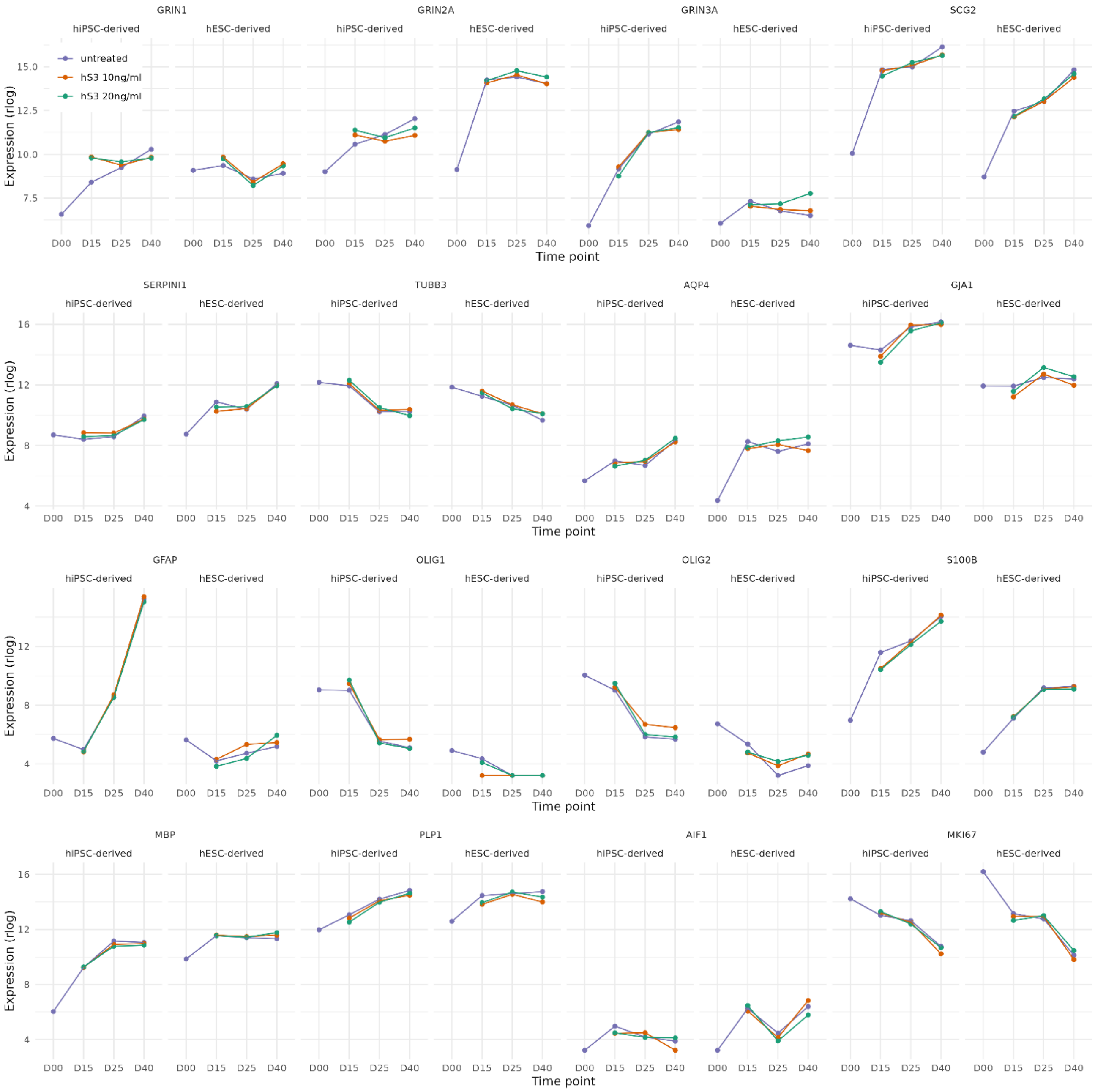
GRIN1
GRIN2A
GRIN3A
SCG2
SERPINI1
TUBB3
AQP4
GJA1
GFAP
OLIG1
OLIG2
S100B
MBP
PLP1
AIF1
MKI67
hiPSC-derived
hESC-derived
untreated
hS3 10ng/ml
hS3 20ng/ml
Expression (rlog)
Time point
D00
D15
D25
D40

E

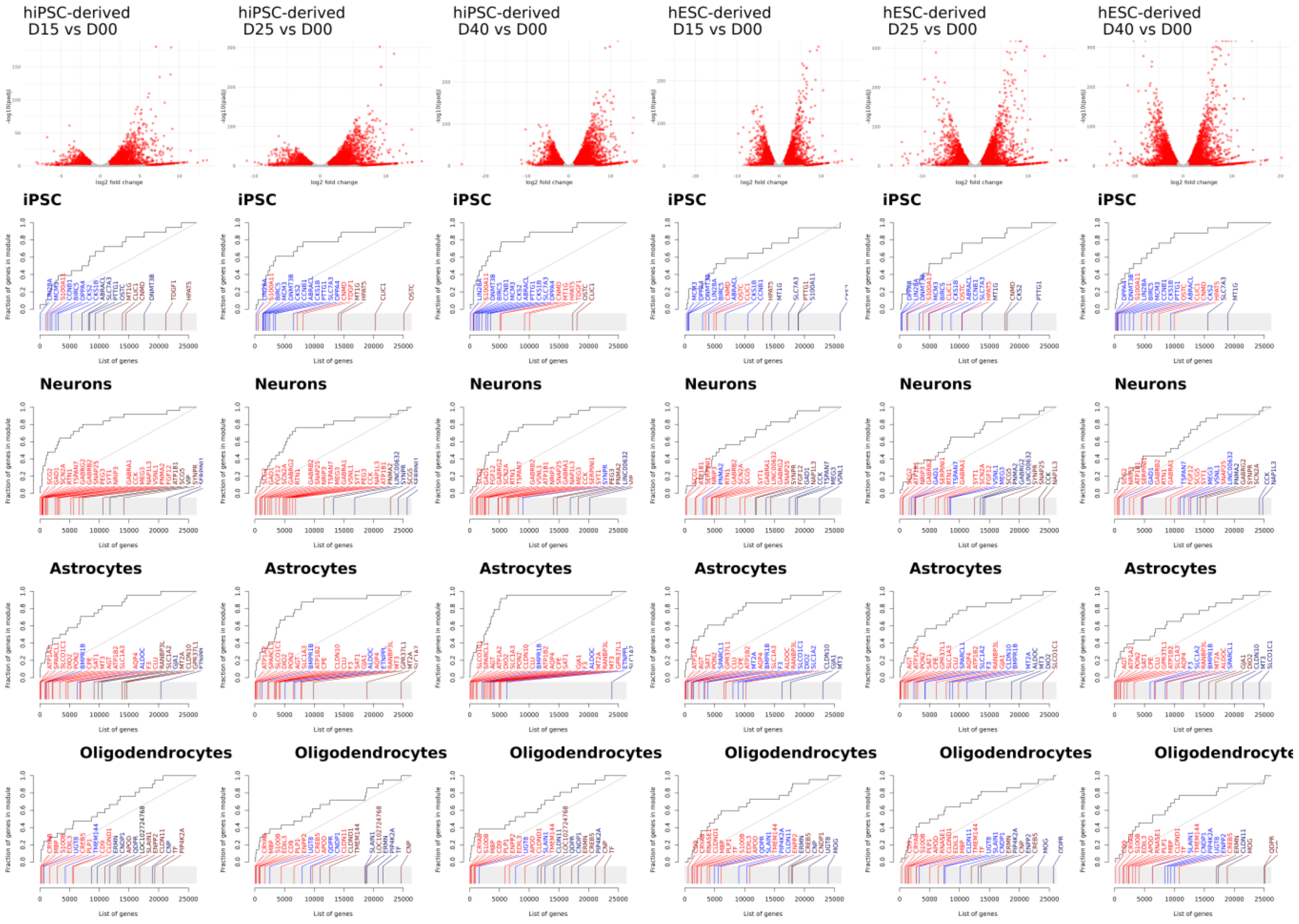

Supplementary Figure 4. (A) Representative Immunofluorescence staining of cell identity markers in untreated and prolonged treated hiPSC-derived and hESC-derived neuronal cultures differentiation day 45, Scalebars =20µm **(B)** Quantification of the proportion of marker-positive area relative to the total image area revealed no statistically significant differences in the expression of cell identity markers (TUBB3, GFAP, OLIG2, AIF1, S100B), the proliferation marker MKI67, or DAPI between treated and untreated groups (n = 4). **(C)** Normalization of marker-positive area to the total DAPI-stained cell area similarly showed no significant differences in the expression of cell identity markers between treated and untreated groups (n = 4). **(D)** RNA-Sequencing confirms no significant expression difference of cell identity marker genes (GRIN1, GRIN2A, GRIN3A, SCG2, SERPINI1, TUBB3, APQ4, GJA1, GFAP, OLIG1, OLIG2, S100B, MBP, PLP1, AIF1) and proliferation marker MKI67 between prolonged treated and untreated hiPSC-derived and hESC-derived neuronal cultures, purple = untreated, orange = prolonged treated with 10ng/ml hS3, green = prolonged treated with 20ng/ml hS3, blue D00 indicates NPC sample of which all three groups are generated from. Plotted is rlog (regularized logarithm transformation) expression at 4 time points D00 = NPC, D15 = Differentiation Day 15, D25 = Differentiation Day 25, D40 = Differentiation Day 40. A rlog > 10 indicates highly expressed genes, a rlog < 5 indicates lowly expressed genes **(E)** Differential Expression during differentiation of hiPSC-derived (left) and hESC-derived (right) neuronal cultures. Upper panel shows differential expressed genes between differentiation day 15 and 0, differentiation

day 25 and 0 and differentiation day 40 and 0 of both cell lines. A differential expressed gene is defined by a Log2 fold change threshold of ≥ 1 and considered significant with p-value threshold of 0,05 labeled in red. Lower panels show timepoint dependent evidence plots for gene set regulation. Both cell lines show downregulation of iPSC gene set and upregulation of neuronal subtype gene sets. Plotted is fraction of genes in module to list of genes. Blue indicates downregulation and red indicates upregulation of that gene compared to the same gene on differentiation day 0. A gene set is considered significantly regulated with an effect size (AUC) ≥ 65 and False Discovery Rate (FDR) ≤ 0,05

Supplementary Figure 5

A

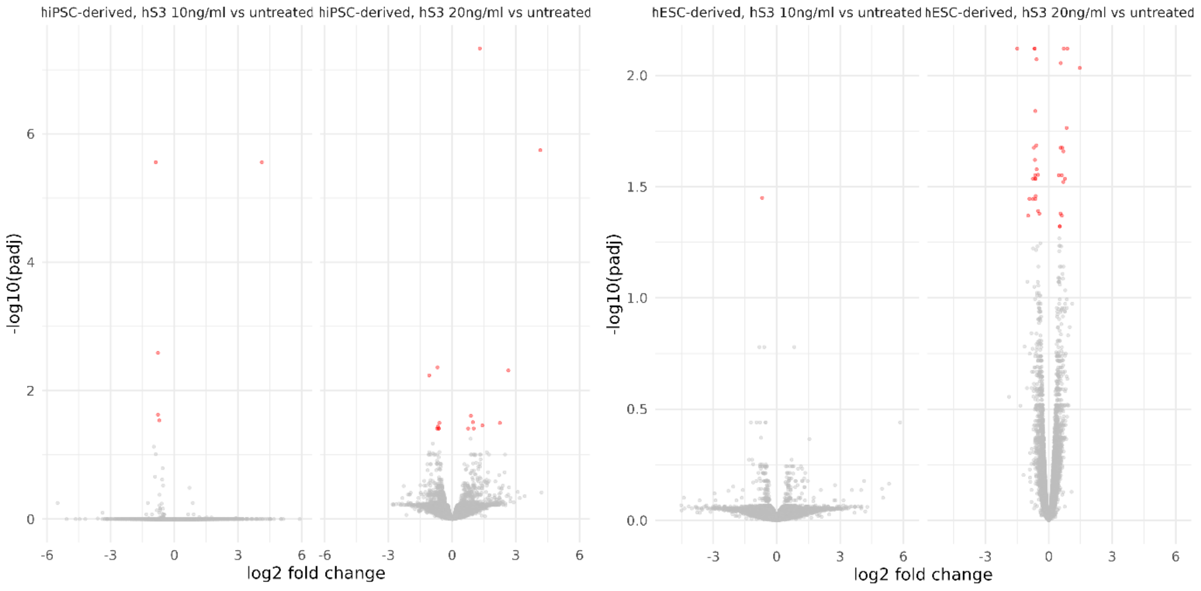

B

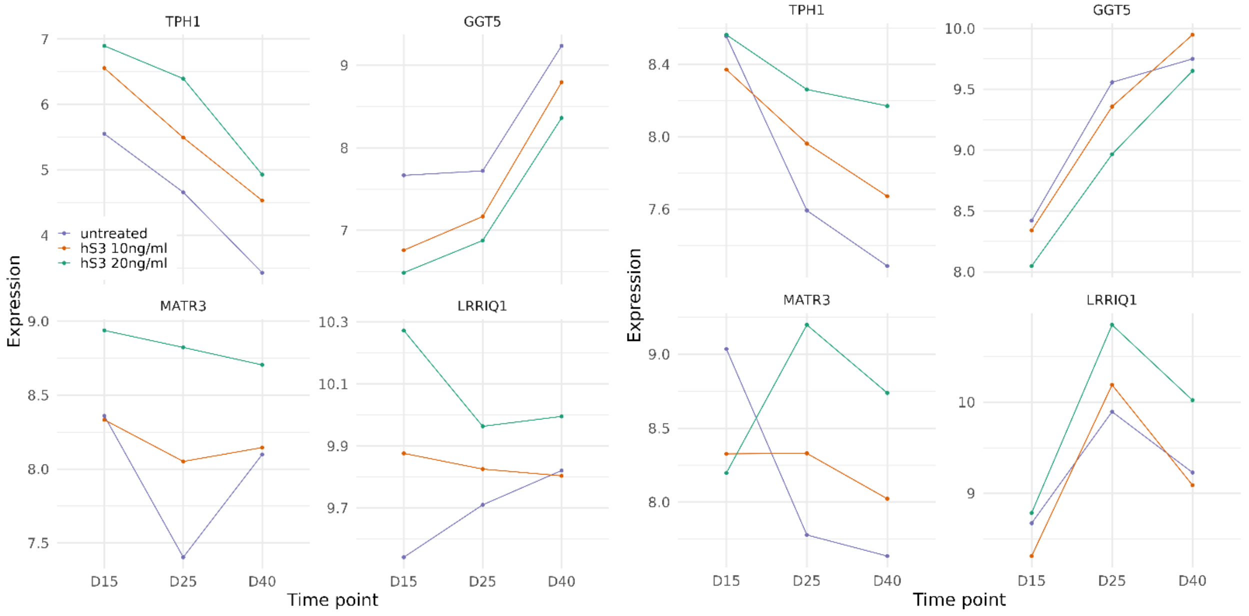

C

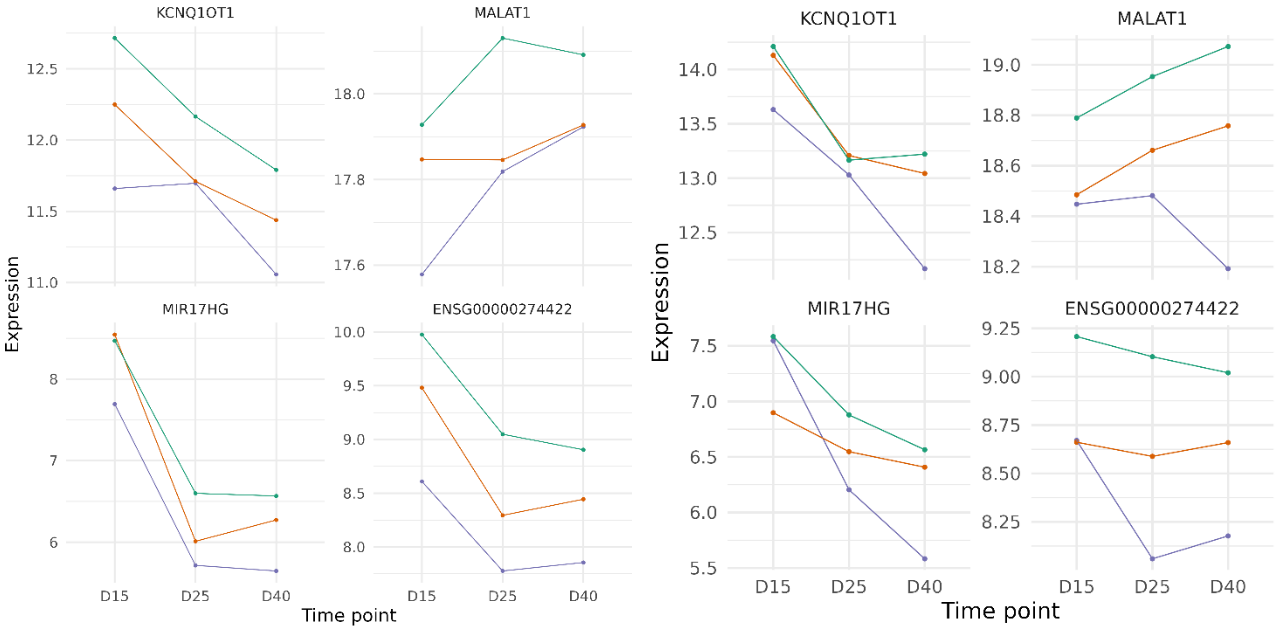

D

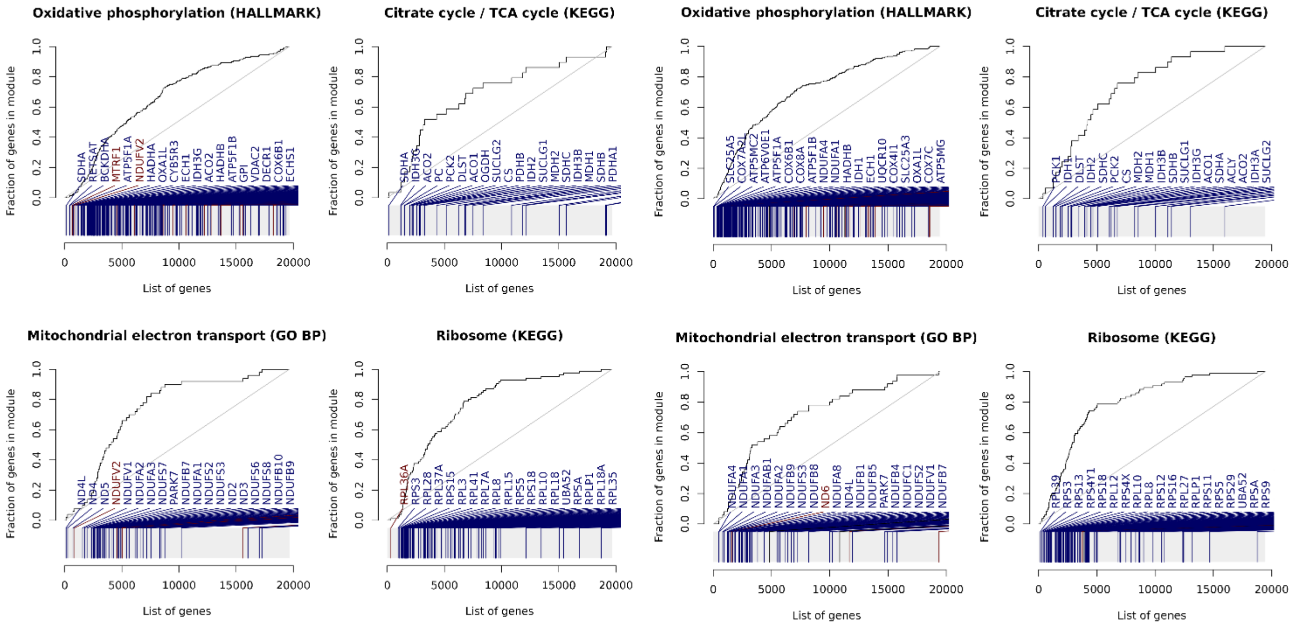

Supplementary Figure 5. Prolonged hS3 treated hiPSC-derived and hESC-derived neuronal cultures show dose dependent differentially expressed apoptosis related genes and dose dependent downregulation of apoptosis related metabolic pathways. **(A)** Volcano plots of differential expressed

genes of hiPSC-derived (left panel) and hESC-derived (right panel) neuronal cultures prolonged treated with 10ng/ml hS3 (left) or 20ng/ml hS3 (right). Plotted are differential expressed genes between prolonged treated with 10ng/ml hS3 and untreated and between prolonged treated with 20ng/ml hS3 and untreated independent of timepoint and plotted as log10(padj) to log2FoldChange. A differential expressed gene is indicated by red color **(B)** examples of dose dependent significant differential expressed apoptosis related genes in both cell line, hiPSC-derived (left panel) and hESC-derived (right panel) **(C)** Examples of dose dependent significant differential expressed apoptosis related LncRNAs in both cell lines. hiPSC-derived (left panel) and hESC-derived (right panel) **(D)** Examples of significant downregulated metabolic pathways in with 20ng/ml hS3 prolonged treated neuronal cultures. Blue indicates downregulated compared to untreated control. hiPSC-derived (left panel) and hESC-derived (right panel) A gene set is considered significantly regulated with an effect size (AUC) ≥ 65 and False Discovery Rate (FDR) $\leq 0,05$. A differentially expressed gene (DEG) is defined by a log2 fold change (LFC) threshold of ≥ 0.5 and is considered significant if it meets a p-value threshold of < 0.001 and an adjusted p-value (padj) threshold of < 0.1, calculated using a false discovery rate (FDR) control of ≤ 0.1.

Supplementary Figure 6

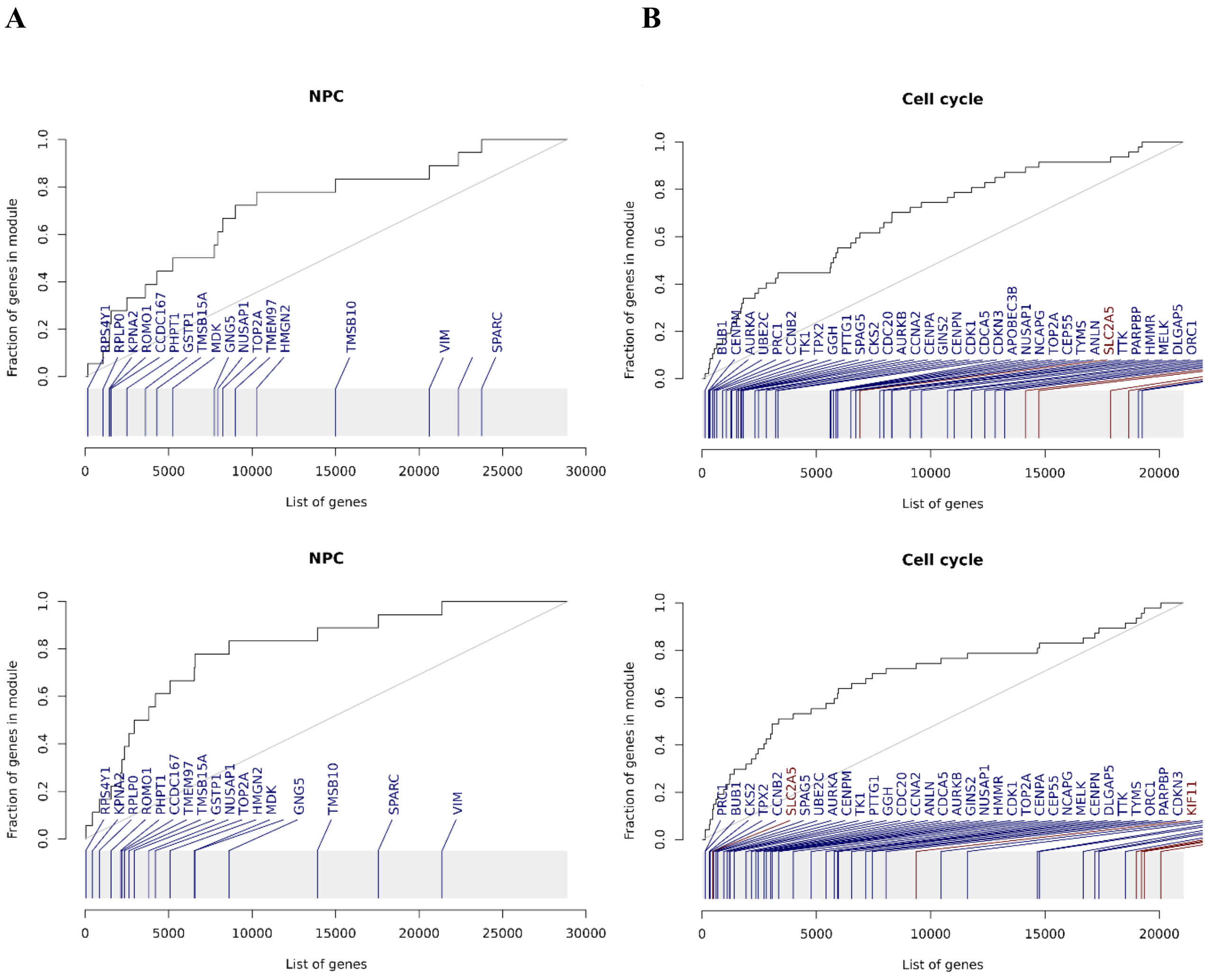

Supplementary Figure 6. Prolonged hS3 treated hiPSC-derived and hESC-derived neuronal cultures show early inhibition of proliferation and cell cycle genes compared to untreated controls. Significant downregulation of NPC gene set **(A)** and Cell Cycle gene set **(B)** in with 10ng/ml hS3 prolonged treated (upper panel) and with 20ng/ml prolonged treated (lower panel) neuronal cultures compared to untreated controls on differentiation day 15. A gene set is considered significantly regulated with an effect size (AUC) ≥ 65 and False Discovery Rate (FDR) ≤ 0,05.
